# Assessing the impact of COVID-19 interventions on influenza-like illness in Beijing and Hong Kong: an observational and modeling study

**DOI:** 10.1186/s40249-023-01061-8

**Published:** 2023-02-16

**Authors:** Xingxing Zhang, Jing Du, Gang Li, Teng Chen, Jin Yang, Jiao Yang, Ting Zhang, Qing Wang, Liuyang Yang, Shengjie Lai, Luzhao Feng, Weizhong Yang

**Affiliations:** 1 School of Population Medicine and Public Health, Chinese Academy of Medical Sciences and Peking Union Medical College, Beijing, 100073 China; 2 Beijing Centre for Disease Prevention and Control, Beijing, 100013 China; 3 Department of Applied Mathematics and Statistics, Stony Brook University, Stony Brook, NY 11794-3600 USA; 4 Department of Management Science and Information System, Faculty of Management and Economics, Kunming University of Science and Technology, Kunming, 650506 China; 5 WorldPop, School of Geography and Environmental Science, University of Southampton, Southampton, SO17 1BJ UK

**Keywords:** Influenza-like illness, Non-pharmaceutical intervention, COVID-19, SARS-CoV-2, Influenza, China

## Abstract

**Background:**

The impact of coronavirus diseases 2019 (COVID-19) related non-pharmaceutical interventions (NPIs) on influenza activity in the presence of other known seasonal driving factors is unclear, especially at the municipal scale. This study aimed to assess the impact of NPIs on outpatient influenza-like illness (ILI) consultations in Beijing and the Hong Kong Special Administrative Region (SAR) of China.

**Methods:**

We descriptively analyzed the temporal characteristics of the weekly ILI counts, nine NPI indicators, mean temperature, relative humidity, and absolute humidity from 2011 to 2021. Generalized additive models (GAM) using data in 2011–2019 were established to predict the weekly ILI counts under a counterfactual scenario of no COVID-19 interventions in Beijing and the Hong Kong SAR in 2020–2021, respectively. GAM models were further built to evaluate the potential impact of each individual or combined NPIs on weekly ILI counts in the presence of other seasonal driving factors in the above settings in 2020–2021.

**Results:**

The weekly ILI counts in Beijing and the Hong Kong SAR fluctuated across years and months in 2011–2019, with an obvious winter-spring seasonality in Beijing. During the 2020–2021 season, the observed weekly ILI counts in both Beijing and the Hong Kong SAR were much lower than those of the past 9 flu seasons, with a 47.5% [95% confidence interval (*CI*): 42.3%, 52.2%) and 60.0% (95% *CI*: 58.6%, 61.1%) reduction, respectively. The observed numbers for these two cities also accounted for only 40.2% (95% *CI*: 35.4%, 45.3%) and 58.0% (95% *CI*: 54.1%, 61.5%) of the GAM model estimates in the absence of COVID-19 NPIs, respectively. Our study revealed that, “Cancelling public events” and “Restrictions on internal travel” measures played an important role in the reduction of ILI in Beijing, while the “restrictions on international travel” was statistically most associated with ILI reductions in the Hong Kong SAR.

**Conclusions:**

Our study suggests that COVID-19 NPIs had been reducing outpatient ILI consultations in the presence of other seasonal driving factors in Beijing and the Hong Kong SAR from 2020 to 2021. In cities with varying local circumstances, some NPIs with appropriate stringency may be tailored to reduce the burden of ILI caused by severe influenza strains or other respiratory infections in future.

**Graphical Abstract:**

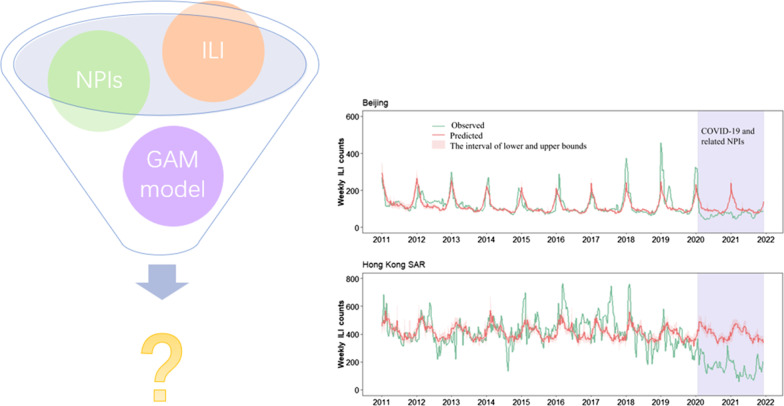

**Supplementary Information:**

The online version contains supplementary material available at 10.1186/s40249-023-01061-8.

## Background

Non-pharmaceutical interventions (NPIs) during the coronavirus diseases 2019 (COVID-19) pandemic have altered not only the spread of SARS-CoV-2, but also the predictable circulation patterns of many other infectious diseases. For instance, influenza viruses and human metapneumovirus have circulated at historic lows through May 2021 in the United States of America (USA) [1]. There was no typical winter surge in hospitalizations related to respiratory syncytial virus among children in 2020 [2]. While respiratory infectious diseases were likely to be the most affected, infectious diseases with other transmission modes, such as gastrointestinal, sexually transmitted, or even vector-borne diseases, may have also been impacted [3]. An example of this is the dramatic reduction in the incidence of norovirus infections in nine US states owing to NPIs [4]. The COVID-19 pandemic and its related NPIs have led to a departure from seasonal disease circulation patterns, and in many locations, the usual circulation of these viruses was absent for more than a year, only to resurge in unexpected ways [5].

Many studies have focused on the impact of NPIs during the COVID-19 pandemic on seasonal influenza worldwide. Reportedly, during the 2019–2020 season, the activity of influenza was largely reduced in both northern and southern China and the USA [6]. Similarly, significantly lower influenza activity was noted with no seasonal influenza outbreaks observed in the Southern Hemisphere in 2020 [7]. Additionally, some studies suggest some international travel-related, personal protective and social distancing NPIs could be considered and reserved for pandemic influenza in the future [8–10]. Although there is consensus that influenza activity decreased sharply after the COVID-19 pandemic in specific countries or regions, the impact of each NPI on ILI remains unclear, especially at the municipal scale. Therefore, it is necessary to explore the effects of NPIs on influenza-like illness (ILI) in the presence of other known seasonal driving factors such as meteorological factors.

Beijing, the capital city of China, is in the temperate region of northern China, whereas the Hong Kong SAR is in a subtropical region. Owing to the unique socioeconomic, climatic, and political characteristics, the influenza circulation pattern during the COVID-19 pandemic in these two areas was closely observed. In this study, we aimed to assess the impact of NPIs on outpatient ILI consultations in Beijing and the Hong Kong SAR from 2020 to 2021 using observational and modeling methods.

## Methods

### Data collection

#### Influenza surveillance data

We collected weekly ILI rates (the proportion of ILI consultations out of all outpatient visits) in Beijing and the Hong Kong SAR from the 1st week of 2011 to the 50th week of 2021. The weekly ILI rates in Beijing were retrieved from a sentinel surveillance system for ILI [11], which was designed and led by the Beijing Center for Disease Control and Prevention (BJCDC) and comprised 421 sentinel hospitals. In this system, outpatient clinicians in internal medicine, the emergency department, fever clinics, and pediatric clinics were required to diagnose all ILI cases using the World Health Organization definition of ILI (patients presenting with fever ≥ 38 °C and cough or sore throat) and to record the number of ILI consultations by age group (0–4, 5–14, 15–24, 25–59, and 60 + years) [12]. These data were then reported daily to the BJCDC via an internet-based system. The weekly ILI rates were obtained by dividing ILI consultations by outpatient visits. This ILI surveillance was conducted throughout the year to monitor influenza virus activity in Beijing.

The weekly ILI rates in the Hong Kong SAR were obtained from the Centre for Health Protection of Hong Kong SAR, based on General Outpatient Clinics/Private Medical Practitioner Clinics sentinel surveillance (Weekly consultation rates of influenza-like illness. https://www.chp.gov.hk/en/static/24015.html. Accessed 1 August 2022.) [13, 14]. The ILI sentinel surveillance network comprised approximately 60 outpatient clinics [15]. At the end of each week, sentinel practitioners reported weekly data on the rates of ILI per 1000 outpatient consultations [16]. Age-specific ILI rates were not reported. The data were retrieved from Hong Kong Island, Kowloon, New Territories East, and New Territories West and aggregated [17]. This ILI surveillance was conducted throughout the year to monitor influenza virus activity in the Hong Kong SAR.

In the Chinese mainland, each surveillance year comprises a 12-month interval; from the 14th week of one year to the 13th week of the following year. For consistency within this study, the surveillance year in the Hong Kong SAR was defined as the same as that in Beijing.

#### COVID-19 NPIs data

We downloaded daily NPIs data from the Oxford COVID-19 Government Response Tracker (OxCGRT; Blavatnik School of Government, University of Oxford, United Kingdom; OxCGRT/covid-policy-tracker-legacy. https://github.com/OxCGRT/covid-policy-tracker-legacy. Accessed 1 August 2022.) [18] in Beijing and the Hong Kong SAR from the 1st week of 2020 to the 50th week of 2021. OxCGRT collects publicly available information on governments’ COVID-19 response [18]. Eight of the policy indicators (C1–C8) relate to containment and closure policies, such as school closures and restrictions on movement. Four of the indicators (E1–E4) concern economic policies, such as income support to citizens or provision of foreign aid. Eight indicators (H1–H8) cover health system policies, such as the COVID-19 testing regimes or emergency investments into healthcare. Three indicators (V1–V3) concern vaccination policies. Finally, a miscellaneous indicator (M1) is for notes that do not fit elsewhere. For each indicator, the OxCGRT scores used a numeric scale with higher scores signifying more intense or wider coverage of the interventions, and data are collected and updated in real-time and reported daily [19].

In this study, nine indicators considered likely to play a key role in influenza transmission based on epidemiological principles and previous studies were retrieved to establish the COVID-19 NPI datasets (Additional file 1: Table S1). Given the potential collinearity between these NPI indicators in the modelling, we combined similar indicators (Additional file 1: Table S2) by calculating their mean. C1 and C2 were combined to C12 representing “closings of schools and workplaces”. C3 and C4 were integrated to C34 representing “cancelling public events or gatherings”. C5, C6 and C7 were combined to C567 representing “restrictions on internal travel”. C1‒8 and H6 were averaged to “All NPIs as a whole” representing the overall intensity of NPIs. Daily NPI data were converted to weekly averages to be consistent with the ILI data.

#### Meteorological data

Daily meteorological data, including mean temperature, mean dew point temperature, and other meteorological factors in Beijing and the Hong Kong SAR, were obtained from the National Centers for Environmental Information (Global Surface Summary of the Day—GSOD. https://www.ncei.noaa.gov/access/search/data-search/global-summary-of-the-day?bbox=53.544,73.620,18.198,134.761&place=Country:194&stations=54511099999&pageNum=4. Accessed 10 August 2022.) [20] for the 1st week of 2011 to the 50th week of 2021. Temperature and humidity have been found to play a more important role in influenza transmission by previous studies [21, 22], which were incorporated into models in the study. The daily saturation vapor pressure* (*$$E$$*)*, daily actual vapor pressure* (*$$e)$$, daily relative humidity *(*$$RH$$*)*, Kelvin temperature *(T)*, and daily absolute humidity* (*$$AH$$*)* were derived from Eqs. 1–5, respectively. In addition, $$t$$ and $${t}_{d}$$ represented mean temperature and mean dew temperature in Eqs. 1–5, respectively. Daily meteorological data were also converted to weekly averages to be consistent with the ILI data.

1 $$\begin{array}{c}E=6.112\mathrm{exp}\left(\frac{17.67*t}{t+243.5}\right)\end{array}$$

2 $$\begin{array}{c}e=6.112\mathrm{exp}\left(\frac{17.67*{t}_{d}}{{t}_{d}+243.5}\right)\end{array}$$

3 $$\begin{array}{c}RH=\frac{e}{E}\times 100\%\end{array}$$

4 $$\begin{array}{c}T=t+273.15^\circ{\rm C} \end{array}$$

5 $$\begin{array}{c}AH=217\times \frac{e}{T}\end{array}$$

#### Public and school holiday data

The holiday data were obtained from the WorldPop Data repository (Global Holiday Data. https://hub.worldpop.org/project/categories?id=19. Accessed 10 August 2022.) [23] and a previous relevant study [24] for the 1st week of 2011 to the 50th week of 2021. The holiday datasets were established as a time series to record how many days in each week contained public or school holidays.

#### Population density data

The yearly population density data of Beijing and the Hong Kong SAR was downloaded from the statistical yearbook from the portal of Beijing Municipal Bureau Statistics (Beijing Statistical Yearbook 2022. https://nj.tjj.beijing.gov.cn/nj/main/2022-tjnj/zk/indexch.htm. Accessed 4 August 2022.) [25] and Census and Statistics Department of the Government of the Hong Kong SAR (Demographic Trends in Hong Kong 1991–2021. https://www.censtatd.gov.hk/sc/EIndexbySubject.html?pcode=B1120017&scode=150. Accessed 4 August 2022.) [26].

### Data analysis

#### Descriptive analysis

**Fig. 1:**
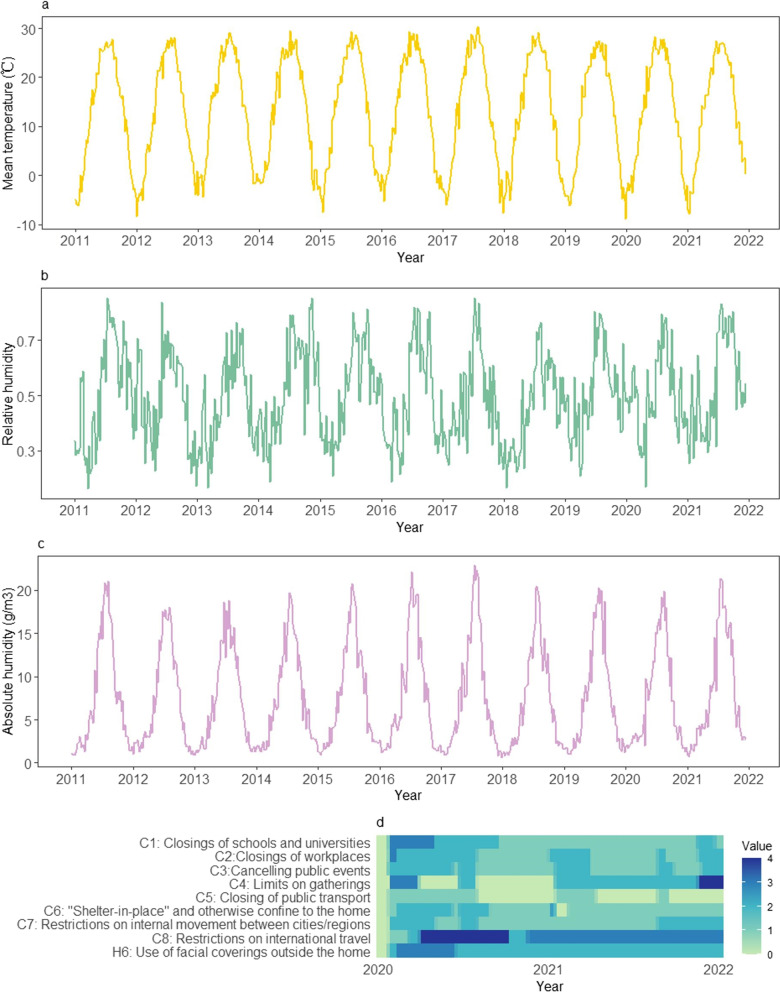
The changes of mean temperature (**a**), absolute humidity (**b**), and relative humidity (**c**) from 2011 to 2021 and the stringency of 9 NPI indicators (**d**) from 2020 to 2021 in Beijing. *NPI* non-pharmaceutical intervention

**Fig. 2:**
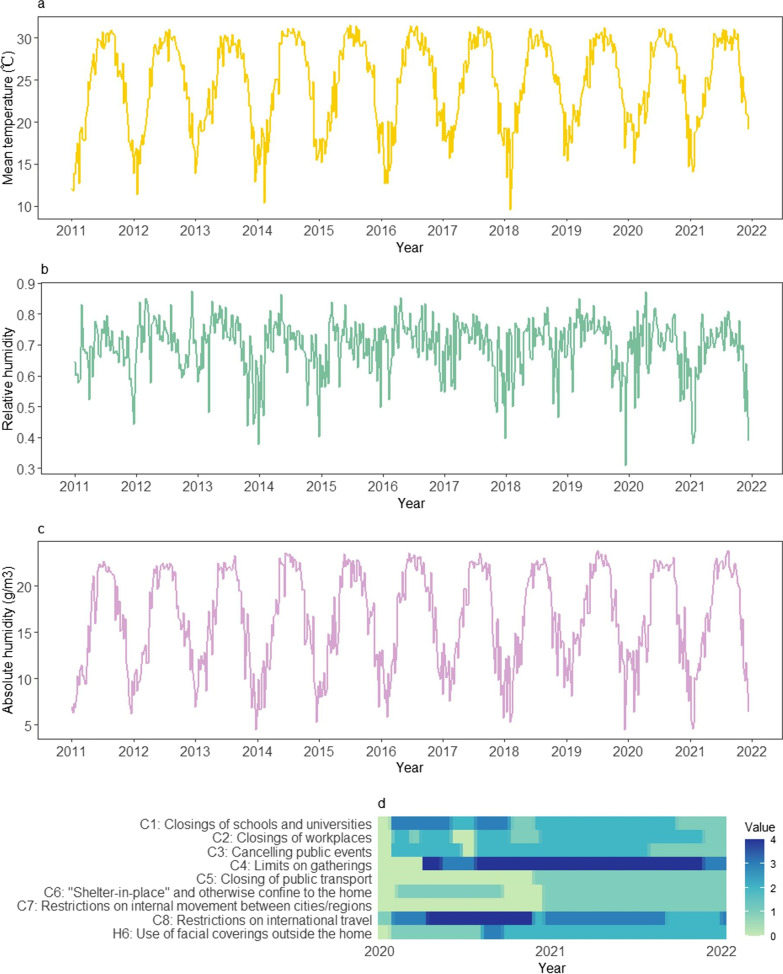
The changes of mean temperature (**a**), absolute humidity (**b**), and relative humidity (**c**) from 2011 to 2021 and the stringency of 9 NPI indicators (**d**) from 2020 to 2021 in the Hong Kong SAR. *NPI* non-pharmaceutical intervention. Hong Kong SAR: Hong Kong Special Administrative Region

We conducted descriptive analysis to present the temporal distribution of the mean temperature, relative humidity, and absolute humidity from the 1st week of 2011 to the 50th week of 2021 in Beijing and the Hong Kong SAR, respectively (Figs. 1 and 2). The nine NPI indicators were descriptively analyzed from the 1st week of 2020 to the 50th week of 2021 in the above two settings (Figs. 1 and 2).

**Fig. 3:**
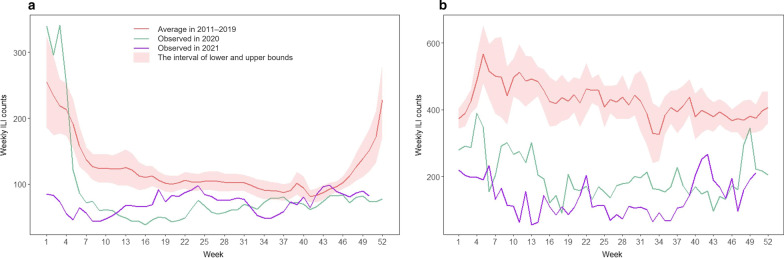
Comparison of the observed weekly ILI counts in 2020–2021 with mean and 95% *CI* of ILI in 2011–2019 in **a** Beijing and **b** the Hong Kong SAR, respectively. *ILI* influenza-like illness, *CI* confidence interval, *Hong Kong SAR* Hong Kong Special Administrative Region

**Fig. 4:**
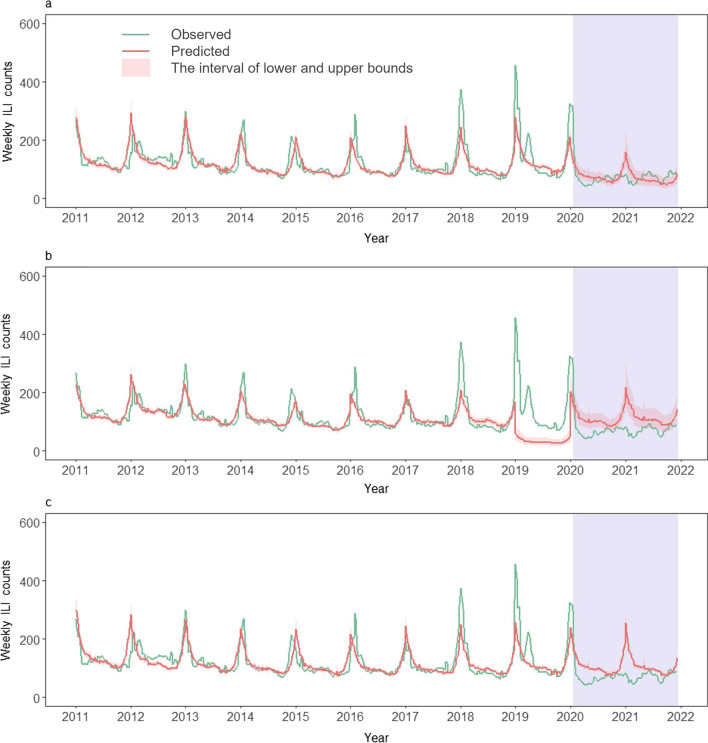
Observed and predicted weekly ILI counts from 2011 to 2021 in Beijing, estimated by GAM models using: **a** Eq. 6 and ILI data in 2011–2019, **b** Eq. 6 and ILI data in 2011–2017, and **c** Eq. 7 and ILI data in 2011–2019, respectively. The estimated ILI in 2020–2021 were predicted under a counterfactual scenario of no COVID-19 interventions. The purple-shaded parts indicate the period of the COVID-19 pandemic and its related NPIs. *ILI* influenza-like illness, *GAM* generalized additive model, *COVID-19* coronavirus disease 2019, *NPIs* non-pharmaceutical interventions

**Fig. 5:**
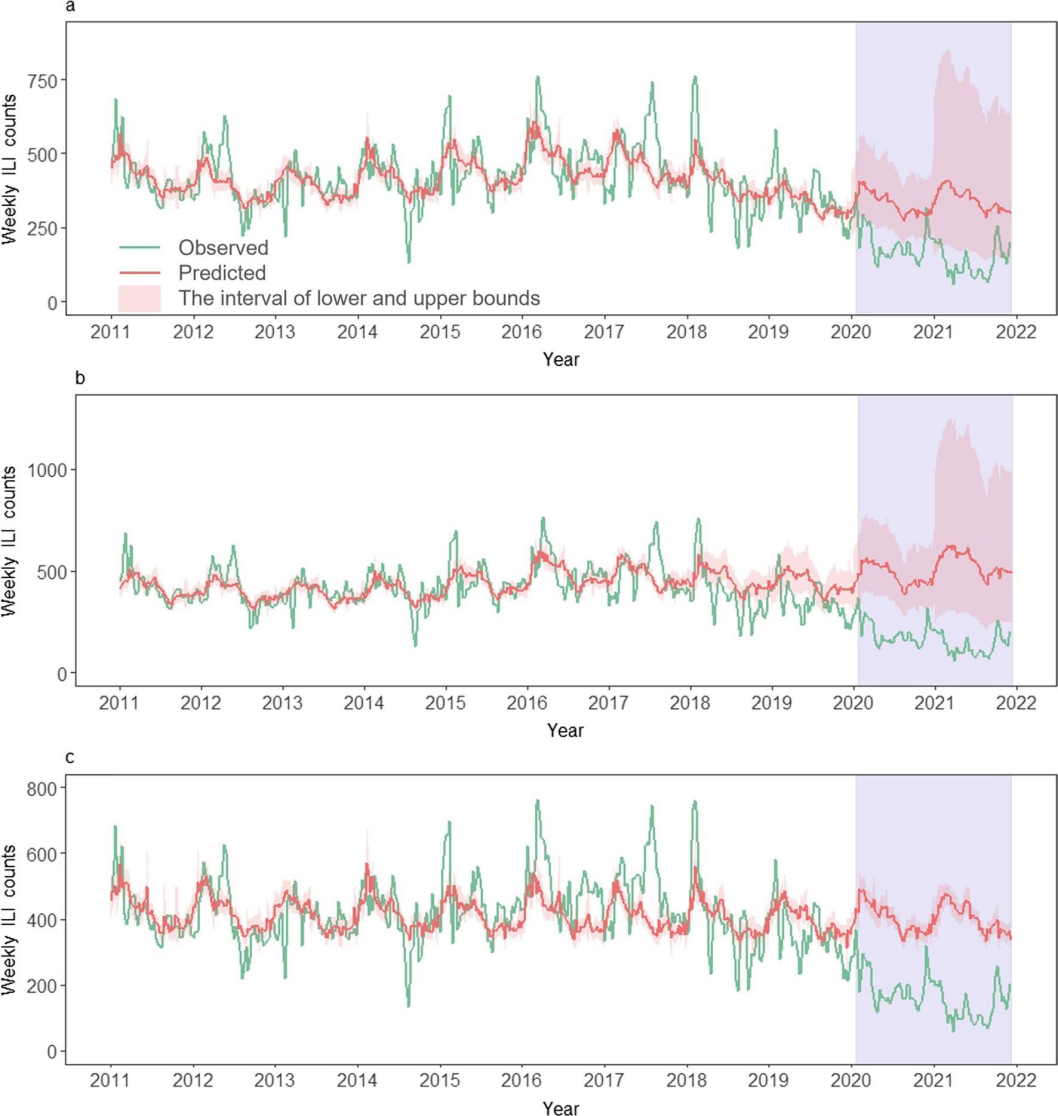
Observed and predicted weekly ILI counts from 2011 to 2021 in the Hong Kong SAR, estimated by GAM models using: **a** Eq. 6 and data in 2011–2019, **b** Eq. 6 and data in 2011–2017, and **c** Eq. 7 and data in 2011–2019, respectively. The estimated ILI in 2020–2021 were predicted under a counterfactual scenario of no COVID-19 interventions. The purple-shaded parts indicate the period of the COVID-19 pandemic and its related NPIs. *ILI* influenza-like illness, *Hong Kong SAR* Hong Kong Special Administrative Region, *GAM* generalized additive model, *COVID-19* coronavirus disease 2019, *NPIs* non-pharmaceutical interventions

The weekly ILI counts were computed by multiplying the weekly ILI rate by a constant 10,000, representing weekly ILI consultations per 10,000 outpatient visits. Given that the incubation period of influenza (1‒4) days [27] and the lagged effect of meteorological factors and NPIs on influenza transmission from exposure to reporting, the weekly ILI counts were smoothen one week back using the moving average method in the study. Then the temporal trend of the average weekly ILI counts in 2011–2019, the observed in 2020, and the observed in 2021 was presented together from the 1st week to the 52nd week in Beijing and the Hong Kong SAR, respectively (Fig. 3). The interannual and seasonal trend of observed weekly ILI counts was also presented together with estimates of the GAM models below from the 1st week of 2011 to the 50th week of 2021 in each city (Figs. 4 and 5).

#### GAM models

First, GAM models based on data from the 1st week of 2011 to the 52nd week of 2019 was established to predict the weekly ILI counts for the COVID-19 period of the 1st week of 2020 to the 50th week of 2021 under a counterfactual scenario of no COVID-19 and its interventions in Beijing and the Hong Kong SAR, respectively. Before modeling, collinearity between mean temperature, relative humidity, and absolute humidity was analyzed using the Pearson correlation method (Additional file 1: Tables S3 and S4). Thus, if the correlation coefficient for a pair was greater than 0.7, only one was included in the model. As a result, mean temperature and relative humidity, but not absolute humidity, were incorporated into two types of predictive GAM models as follows. Equation 6 takes both seasonal and interannual variations into account, which Eq. 7 only considers seasonal features.

6 $$\begin{array}{c}\mathit{log}\left[E\left({Y}_{i}\right)\right]=\alpha + ns\left({Year}_{i}, df\right)+ns\left({W}_{i}, df\right) + ns\left({T}_{i}, df\right)\\ +ns\left({RH}_{i},df\right) + factor\left({H}_{i}\right)+pd\end{array}$$

7 $$\begin{array}{c}\mathit{log}\left[E\left({Y}_{i}\right)\right]=\alpha + ns\left({W}_{i}, df\right) + ns\left({T}_{i}, df\right)\\ +ns\left({RH}_{i},df\right) + factor\left({H}_{i}\right)+pd\end{array}$$

where *E(Y*_*i*_*)* is the expected weekly ILI counts in a given week (*i)*, and the link function here is *log()* with the assumption that weekly ILI counts follows log normal distribution; α is the intercept; $ns\left(.\right)$ is a cubic spline function, $df$ is the degree of freedom; ${Year}_{i}$ is a time series of year numbers (1, 2, 3…, 11) in the dataset, representing the potential interannual long-term trend in ILI counts; ${W}_{i}$ is a time series of week numbers (1, 2, 3…, 52) in a calendar year, representing the potential seasonality trend in weekly ILI counts; ${T}_{i}$ is mean temperature in week *(i)*; ${RH}_{i}$ is relative humidity in week *(i)*. The degrees of these factors are determined by partial autocorrelation functions [28]. ${H}_{i}$ is an indicator variable that equals 0–7 representing how many days of school and public holidays in a week (*i)*. The population density (*pd*) of each city per year was also incorporated into the model to adjust for potential demographic confounding factors across space and time.

The models were built and trained using 2011–2019 data in Beijing and the Hong Kong SAR, respectively. The goodness of fit of the GAM models was assessed by root mean square error (RMSE), Akaike information criterion (AIC), and adjusted R-square (Additional file 1: Tables S5 and S6). However, the ILI in 2018–2019 in the Hong Kong SAR were much lower than those in the previous years, which might be due to the instability of ILI reporting during the protests or riots in the city during 2018–2019. Thus, the inclusion of the ${Year}_{i}$ term or ILI data in 2018–2019 could lead to a continuous downward trend in ILI prediction from 2018 to 2021, which might not be fully representative of the real situation. To address this, we also tested and compared Eq. 6 using 2011–2017 data for both cities. Considering the data reliability, the goodness of fit of the models and the uncertainty of predictions (Figs. 4 and 5), the subsequent results in the main text were mainly retrieved from the predictions of Eq. 7, while the results of other models were presented in the Additional file 1.

Second, a simple GAM model including univariate interventions was established to assess the relationship between the relative reduction of weekly ILI counts and each individual or combined NPI measure, from 4th week of 2020 when the large-scale COVID-19 NPIs were implemented, to the 50th week of 2021 in Beijing and the Hong Kong SAR, respectively. After examining the collinearity using the Pearson correlation (Additional file 1: Tables S7 and S8), the relative change of mean temperature and the relative change of relative humidity, but not the relative change of absolute humidity, were incorporated into the model. The basic model is as follows:

8 $$\begin{array}{c}E\left({Y}_{i}\_c\right)= a\_c + {{\beta }_{j}X}_{j}+ns\left({S}_{i}, df\right) + ns\left({T}_{i}\_c, df\right)\\ + ns\left({RH}_{i}\_c,df\right) + {pd}_{i}\_c\end{array}$$

where $E\left({Y}_{i}\_c\right)$ is the expected relative reduction of ILI counts in week (i) (Eq. 9) with the assumption that the relative reduction of weekly ILI counts follows normal distribution; ${a}_{c}$ is the intercept; ${S}_{i}$ is a time series of week numbers (1,2,3…,99) during the study period in 2020–2021, representing the potential seasonality and long-term trend in weekly ILI counts; ${T}_{i}\_c$ is the relative change of mean temperature in week (*i*) (Eq. 10); ${RH}_{i}\_c$ is the relative change of relative humidity in week (*i)* (Eq. 11); ${pd}_{i}\_c$ is the relative change of population density (Eq. 12); ${X}_{j}$ refers to the each individual or combined NPI indicator, and ${\beta }_{j}$ is its coefficient. A positive coefficient represents that the intervention, or its intensity might not be statistically associated to the decrease of weekly ILI counts across the whole study period, while a negative coefficient reveals that the implementation of COVID-19 NPIs might be associated with the reduction of ILI in the city.

9 $$\begin{array}{c}{Y}_{i}\_c=\frac{Observed \,weekly \,ILI \,counts-predicted \,weekly \,ILI \,counts}{predicted \,weekly \,ILI \,counts}\end{array}$$

10 $$\begin{array}{c}{T}_{i}\_c=\frac{Observed \,mean \,temperature-average \,in \,the \,past \,9 \,years}{average \,in \,the \,past \,9 \,years \,in \,2011-2019}\end{array}$$

11 $$\begin{array}{c}{RH}_{i}\_c=\frac{Observed \,relative \,humidity-average \,in \,the \,past \,9 \,years}{average \,in \,the \,past \,9 \,years \,in \,2011-2019}\end{array}$$

12 $$\begin{array}{c}pd\_c=\frac{Population \,density-average \,in \,the \,past \,9 \,years}{average \,in \,the \,past \,9 \,years \,in \,2011-2019}\end{array}$$

Third, a multivariate GAM model including multiple interventions was further built to assess the relationship between the relative reduction of weekly ILI counts and the NPI indicators. Due to the collinearity among the individual or combined NPI indicators (Additional file 1: Tables S9–S12), for each individual NPI, only C3, C4, C6, C7, C8, and H6 were incorporated into the GAM model for Beijing and C1, C2, C3, C4, C6, C7, C8, and H6 were incorporated into the model for Hong Kong; for the combined NPI indicators, C34 and C567 were incorporated into the GAM model with C8 and H6 for Beijing and the C12, C34, C567, C8 and H6 were incorporated into the GAM model for Hong Kong. The basic model is as follows:

13 $$\begin{array}{c}E\left({Y}_{i}\_c\right)= \alpha + \sum {\beta }_{j}\left({X}_{j}\right)+ns\left({S}_{i}, df\right) + ns\left({T}_{i}\_c, df\right)\\ + ns\left({RH}_{i}\_c,df\right) + pd\_c\end{array}$$

The meaning of the symbols in Eq. 13 was the same as that in Eq. 8. The goodness of fit of the GAM models in Beijing and the Hong Kong SAR in 2020–2021 was also assessed by RMSE, AIC and adjusted R-square (Additional file 1: Table S13).

#### Cross-validation

To understand the robustness of GAM models, we performed a blocked cross-validation analysis. For the GAM models in 2011–2019, in the first step, data in 2011–2016 (312 consecutive weeks) were used as a training set to establish the model and data in the following year 2017 (52 consecutive weeks) were used as a test set. In the second step, it was pushed forward for a week both for the training and test set. Such analysis continued until the last week of 2019. At last, 105 analyses were performed for each model and goodness of fit indicators (mean and 95% *CI*) were calculated (Additional file 1: Tables S5 and S6). For the GAM models in 2011–2017, in the first step, data in 2011–2015 (260 consecutive weeks) were used as a training set to establish the model and data in the following year 2016 (52 consecutive weeks) were used as a test set. In the second step, it was pushed forward for a week both for the training and test set. Such analysis continued until the last week of 2019. At last, 52 analyses were performed for each model and goodness of fit indicators (mean and 95% *CI*) were calculated (Additional file 1: Tables S5 and S6). Similarly, to test the performance of GAM models for revealing the NPI impacts in 2020–2021, data in the first 52 consecutive weeks were initially used as a training set to establish the model and data in the following consecutive 8 weeks were used as a test set. Both sets were moved forward 1 week at a time in the following steps until the last week of 2021. At last, 35 analyses were performed for each model with the goodness of fit indicators calculated (Additional file 1: Table S13). Inferred from the blocked cross-validation analysis (Additional file 1: Tables S5, S6 and S13), the performance of model 1, 2 and 3 in each block were quite stable (relatively small values of average RMSE, AIC and adjusted R^2^ with narrow 95% confidence intervals, respectively). These further implied that all models were robust to the changes in data and had a low risk of overfitting.

Dataset establishment and data analyses for this study were implemented using R version 4.0.0 (2020-04-24) (R Foundation for Statistical Computing, Vienna, Austria) and Excel Microsoft 365MSO (207 Build 16.0.15427.20182) (Microsoft, Washington, USA).

## Results

### Descriptive analysis

In Beijing, mean temperature exhibited regular seasonal fluctuations, ranging between -9 and 30 degrees (Fig. 1a). The range of relative humidity was 0.1–0.9 (Fig. 1b). The absolute humidity also presented seasonal characteristics, fluctuating between 0 g/m^3^ and 23 g/m^3^ (Fig. 1c). Compared with Beijing, the Hong Kong SAR also showed seasonal fluctuations in temperature and humidity, but the mean temperature was higher, ranging between 9 and 32 degrees (Fig. 2a). Hong Kong was also more humid with the relative humidity of 0.3–0.9 (Fig. 2b) and the absolute humidity between 4 g/m^3^ and 24 g/m^3^ (Fig. 2c). The stringency and duration of the nine NPI indicators for COVID-19 in both cities changed over time in 2020–2021 (Figs. 1d and 2d). In addition, during 2020–2021 season (from 14th week in 2020 to 13th week in 2021, as a surveillance year), the observed weekly ILI counts were much lower than that of the past nine seasons of 2011–2019 in Beijing (Fig. 3a), with a 47.5% (95% *CI*: 42.3%, 52.2%) reduction on the weekly averages. Similarly, the observed weekly ILI counts for the same period in the Hong Kong SAR were much lower than those of past nine seasons (Fig. 3b), with an average of 60.0% (95% *CI*: 58.6%, 61.1%) reduction from 2011‒2019.

### Predicting weekly ILI counts with no COVID-19 NPIs in 2020–2021

In Beijing, annual ILI counts peaked during the colder winter-spring months in 2011–2019 (Fig. 4). Since late January 2020, the ILI counts had reduced substantially and remained at low levels through 2021, with no “winter peaks” (Fig. 4). However, the weekly ILI counts predicted by different models under a counterfactual scenario of no COVID-19 interventions would have a typical winter-spring epidemic season in 2020–2021 (Fig. 4). The averages of observed weekly ILI counts were much lower than predicted average weekly ILI counts during the 2020–2021 season, with a 40.2% (95% *CI*: 35.4%, 45.3%) reduction from predictions (Fig. 4c).

In the Hong Kong SAR, year-round ILI reports with highly irregular fluctuations were also observed across years in 2011–2019 (Fig. 5). Since 2020, the ILI counts had a steep decrease and remained at low levels through 2021, but two peaks could be still observed in late 2020 and 2021 (Fig. 5). The predicted weekly ILI counts using different modelling approaches and historical data were well above the observed values across years in 2020‒2021 (Fig. 5). The observed weekly ILI counts in 2020–2021 season had a 58.0% (95% *CI*: 54.1%, 61.5%) reduction, compared with estimates under the scenario without COVID-19 NPIs (Fig. 5c).

### Potential impacts of individual and combined NPIs on ILI reduction

Inferred from the models of univariable analysis by including one type or group of interventions at a time, each individual or combined NPIs and the overall effect of NPIs were associated with the decrease of the ILI in Beijing (Table 1). Inferred from the coefficients of GAM models using multivariate analysis of interventions, the integration of “Cancelling public events” (− 0.159, 95% *CI*: − 0.220, − 0.099) and “Restrictions on internal travel” (− 0.127, 95% *CI*: − 0.203, − 0.051) played an important role in the reduction of ILI in the city (Table 2). However, only “Restrictions on international travel” (− 0.132, 95% *CI:* − 0.187, − 0.077) were statistically associated with ILI reduction in the Hong Kong SAR, and it seems that the stringency of other measures might not be sufficient to suppress ILI or its variation of implementation across all weeks in 2020‒2021 might not be consistently associated with the decrease of ILI counts during the study period (Tables 1 and 2). The potential impacts of individual and combined NPIs on ILI reduction were also assessed by predicted values from the Eq. 6 using data in 2011–2019 and 2011–2017, respectively, with findings similar to those described above (Additional file 1: Tables S14–S17). In addition, details on the estimated and fitted relative reduction of weekly ILI counts in Beijing and Hong Kong SAR in 2020–2021 using equations 6 and 7 could be available from Additional files 2, 3, 4, 5, 6, 7.

**Table 1:** The potential impact of each individual and combined NPI on weekly ILI counts in Beijing and the Hong Kong SAR, 2020–2021

Interventions	Beijing	Hong Kong SAR
C1: Closings of schools	− 0.086 (− 0.158, − 0.014)*	0.053 (0.008, 0.099)*
C2: Closings of workplaces	− 0.128 (− 0.188, − 0.069)*	0.059 (0.021, 0.097)*
C3: Cancelling public events	− 0.178 (− 0.234, − 0.121)*	0.004 (− 0.043, 0.051)
C4: Limits on gatherings	− 0.046 (− 0.074, − 0.018)*	− 0.001 (− 0.036, 0.035)
C5: Closing of public transport	− 0.096 (− 0.177, 0.015)− *	0.426 (0.272, 0.579)*
C6: Staying in place or at home	− 0.081 (− 0.139, − 0.022)*	0.186 (0.090, 0.282)*
C7: Restrictions on internal travel	− 0.166 (− 0.224, − 0.108)*	0.139 (0.000, 0.278)
C8: Restrictions on international travel	0.059 (0.002, 0.115)*	− 0.113 (− 0.161, − 0.065)*
H6: Mask wearing outside the home	− 0.145 (− 0.270, − 0.021)*	0.071 (0.003, 0.139)*
C12: Closings of schools and workplaces	− 0.168 (− 0.245, − 0.091)*	0.062 (0.018, 0.107)*
C34: Cancelling public events or gatherings	− 0.099 (− 0.140, − 0.057)*	0.005 (− 0.050, 0.059)
C567: Restrictions on internal movement	− 0.088 (− 0.118, − 0.057)*	0.419 (0.246, 0.592)*
All NPIs as a whole	− 0.282 (− 0.378, − 0.186)*	0.079 (− 0.018, 0.177)

The numbers presented are the coefficient with its 95% *CI* (data in the round bracket) of each intervention in GAM models (Eq. 8), to reflect their potential impact on weekly ILI counts. A positive coefficient represents that the intervention, or its intensity might not be statistically associated to the decrease of weekly ILI counts across the whole study period, while a negative coefficient represents the implementation of COVID-19 NPIs might be associated with the reduction of ILI in the city. Besides, population density change and non-parametric terms of s(time), s(mean temperature change), and s(relative humidity change) were also incorporated into the GAM models to adjust for potential confounding factors between cities. *NPI* non-pharmaceutical intervention, *ILI* influenza-like illness, Hong Kong SAR Hong Kong Special Administrative Region, *GAM* generalized additive model. *CI*s, Confidence intervals; COVID-19, Coronavirus disease 2019. *P < 0.05

**Table 2:** Multivariable analysis for the potential impact of NPIs on weekly ILI counts in Beijing and the Hong Kong SAR, 2020–2021

Interventions	Beijing	Hong Kong SAR
*For individual NPI indicators*		
+ C1: Closings of schools	–	− 0.046 (− 0.111, 0.020)
+ C2: Closings of workplaces	–	0.092 (0.027, 0.158)
+ C3: Cancelling public events	− 0.159 (− 0.220, − 0.099)*	− 0.023 (− 0.081, 0.036)
+ C4: Limits on gatherings	0.041 (0.099, 0.006)*	− 0.025 (− 0.065, 0.015)
+ C5: Closing of public transport	–	–
+ C6: Staying in place or at home	− 0.046 (− 0.099, 0.006)	0.140 (− 0.005, 0.285)
+ C7: Restrictions on internal travel	− 0.127 (− 0.203, − 0.051)*	− 0.002 (− 0.220, 0.216)
+ C8: Restrictions on international travel	0.030 (− 0.020, 0.080)	− 0.132 (− 0.187, − 0.077)*
+ H6: Mask wearing outside the home	− 0.101 (− 0.207, 0.004)	0.002 (− 0.081, 0.086)
*For combined NPI indicators*		
+ C12: Closings of schools and workplaces	–	0.036 (− 0.024, 0.097)
+ C34: Cancelling public events or gatherings	− 0.041 (− 0.095, 0.014)	− 0.009 (0.071, 0.052)
+ C567: Restrictions on internal movement	− 0.068 (− 0.106, − 0.031)*	0.297 (0.095, 0.499)
+ C8: Restrictions on international travel	− 0.008 (− 0.061, 0.045)	− 0.115 (− 0.170 − 0.061)*
+ H6: Mask wearing outside the home	− 0.068 (− 0.183, 0.047)	0.001 (− 0.075, 0.076)

The numbers presented here are the coefficient (95% *CI*) of the GAM models (Eq. 13), to reflect the potential impact of synthetical COVID-19 interventions on weekly ILI counts. A positive coefficient represents a potential effect of increasing the weekly ILI counts. A negative coefficient represents a potential effect of reducing the weekly ILI counts. Besides the above NPIs indicators, non-parametric terms of s(time), s(mean temperature change), s(relative humidity change) and population density change were also incorporated into the GAM models. *NPI* non-pharmaceutical intervention, *ILI* influenza-like illness, *Hong Kong SAR* Hong Kong Special Administrative Region, *GAM* generalized additive model, *CI*s Confidence intervals, *COVID-19* Coronavirus disease 2019. –, not incorporated into the model due to the multicollinearity. *P < 0.05

## Discussion

Our study found that the weekly ILI counts decreased and remained at low levels in both Beijing and the Hong Kong SAR from 2020 to 2021. The results of our study are consistent with those of previous similar studies in China and abroad [29–32]. The rapid decrease of ILI counts during the early stage of the COVID-19 pandemic and the sustained low-level activity could largely be attributed to the COVID-19 pandemic and its related NPIs. As ILI is a proxy for influenza activity, a decrease in ILI could also be reasonably interpreted as a decrease in the activity of influenza or other respiratory infections. A possible mechanism may be that NPIs reduce human-to-human contact (e.g., social distancing), interrupt the spread (e.g., travel restrictions), and improve personal hygiene (e.g., face masking and hand washing) to contain or mitigate the transmission, like COVID-19 [8–10]. It has long been recognized that domestic and international travel likely play an important role in the spread of the influenza virus [33]. Thus, the greatly reduced regional and international travel during the pandemic was likely a key factor. Restrictions on gatherings or public events added to the effect. Additionally, students are believed to play a crucial role in influenza transmission within communities, a factor that was eliminated by the closing of schools. Furthermore, mask-wearing has already been proven to effectively reduce the transmission of respiratory viruses, including influenza and SARS-CoV-2. Other reasons behind the steep decrease of ILI could be the lower health care seeking rate during the COVID-19 pandemic [34] or the interruption of routine influenza surveillance by COVID-19.

The performance of GAM models in 2011**–**2019 for Beijing seems better than that for the Hong Kong SAR. Climate factors might partially account for this phenomenon. Beijing is in a temperate zone, where influenza and other common respiratory viruses normally presented a regular seasonal pattern before the COVID-19 pandemic. For example, the influenza virus was primarily transmitted and peaks during the colder winter-spring months. However, in the Hong Kong SAR, seasonal characteristics of influenza are more diverse. Influenza epidemics in the Hong Kong SAR can persist year-round, with two or more peaks occurring in 1 year [35]. The complication of the inherent periodicity and local transmission might make it difficult to simulate and forecast influenza activity in the Hong Kong SAR. In addition, the instability in ILI data reporting in the Hong Kong SAR, e.g., decreasing ILI level in 2018**–**2019 that might be partially attributed to social unrest, added to the uncertainty in the estimates of the GAM models.

Additionally, inference from the GAM model, “Cancelling public events” and “Restrictions on internal travel” measures played an important role in the reduction of ILI in Beijing, whereas “Restrictions on international travel” had a greater impact in the Hong Kong SAR. The unique socio-economic features of the two cities could be the underlying reasons. “Canceling public events” is one of the social-distancing measure, which could significantly reduce person-to-person contact with high intensity [36], especially in a super developed city, like Beijing. It was reported that in Beijing, the average one-way commute is 11.3 kilometers and 48 minutes, with heavily burden of super-commuting, ranking first in China [37, 38]. Thus, “Restrictions on internal travel” might massively reduce movements of crowds, in turn, decreasing the ILI level and influenza activity. The Hong Kong SAR is highly connected with the Chinese mainland and other regions worldwide, attracting over 50 million visitors per year before the pandemic, with a high population density of 6810 per km^2^ [26]. This might increase the importation rate of influenza strains and further facilitate local transmission [15, 35]. Therefore, restrictions on international movements could lead to a decrease in ILI and influenza activity. It should be noted that many measures (e.g., restriction on internal travel) in the Hong Kong SAR were not statistically significant across the whole study period, which does not mean that they have no effect on the prevention and control of respiratory infectious diseases. In addition, the reason might be that the intensity of some NPIs in Hong Kong was not high enough compared to same measures in other regions [39], or the implementation of NPIs was not well documented in the dataset and the multicollinearity of NPIs exists. As to why “International travel restriction” was not significant in Beijing might be partially attributed to the following reasons. First, flights from abroad had been reduced massively in Beijing and diverted strictly to other provinces/cities early since late January 2020 and kept through 2021 and even longer. Second, international travel only accounted for a small proportion of all people into Beijing as travel from other provinces in the Chinese mainland into Beijing were considered as internal movement. Third, the effects of other NPIs (e.g., “Cancelling public events” and “Restrictions on internal travel”) were higher in Beijing and the impact of “International travel restriction” was masked in the modelling.

Our study had several limitations. First, the heterogeneity in the effects of NPIs on ILI counts between the two settings might be associated with other sociodemographic factors, including age structure and influenza vaccination coverage, which were not included in the current study. Second, “mean” was used when combining the NPI indicators because nine indicators (C1‒C8 and H6) used in the study were coded using a numeric scale. However, if the values of indicators were not normally distributed, and the averaged values might lead to biases in the analyses. Third, it was an observational study. Evaluation of the impact of NPIs on ILI counts should be interpreted as an association, rather than a causal correlation. Decreased influenza activity could also be partly attributed to changes in healthcare-seeking behavior or potential disruption of COVID-19 pandemic sentinel surveillance. Fourth, owing to data availability issues, our study period was limited to 2020 through 2021, and the study settings were limited to Beijing and the Hong Kong SAR. Longer time-series data and more representative settings and datasets should be considered to further explore the relationship between ILI activity and NPIs.

However, the COVID-19 pandemic interruption has added uncertainty to the transmission of influenza or other respiratory infections in the future. Although many ILI among populations were prevented by COVID-19 NPIs, as founded in this study, decreased exposure to the respiratory pathogens, e.g., influenza viruses, created an immunity gap as susceptible individuals who avoided infection lack immunity against future infections [5]. Moreover, decreased influenza vaccination has been observed among different populations at multiple locations [40–42], which would also contribute to the immunity gap. As the COVID-19 pandemic evolves, NPIs are becoming more relaxed in more countries and regions progressively. Health systems and society must prepare for the potential non-typical rebound or outbreak of influenza and other respiratory infectious diseases.

## Conclusions

Our study suggests that COVID-19 NPIs had an impact on reducing outpatient ILI consultations in the presence of other seasonal driving factors in Beijing and the Hong Kong SAR from 2020 to 2021. As immunity wanes among populations, potential rebounds or outbreaks of influenza and other respiratory pathogens need to be closely monitored. Based on our findings, some NPIs with appropriate stringency in cities with varying local circumstances may be tailored to reduce the burden of ILI caused by severe influenza strains or other respiratory infections in future.

## Supplementary Information

**Additional file 1: Table S1**. Individual COVID-19 NPI indicators, definition, and coding in Beijing and the Hong Kong SAR.** Table S2**. Combined COVID-19 NPI indicators, definition, and coding in Beijing and the Hong Kong SAR.** Table S3**. Collinearity analysis between meteorological factors in Beijing from 2011 to 2019 using the Pearson correlation method.** Table S4**. Collinearity analysis between meteorological factors in the Hong Kong SAR from 2011 to 2019 using the Pearson correlation method. **Table S5**. The goodness of fit of the predictive GAM models in Beijing with blocked cross-validation method.** Table S6**. The goodness of fit of the predictive GAM models in the Hong Kong SAR with blocked cross-validation method.** Table S7**. Collinearity analysis between relative change of meteorological factors in Beijing from 2020 to 2021 using the Pearson correlation method.** Table S8**. Collinearity analysis between relative change of meteorological factors in the Hong Kong SAR from 2020 to 2021 using the Pearson correlation method.** Table S9**. Collinearity analysis between 9 NPIs indicators in Beijing from 2020 to 2021 using the Pearson correlation method. Table** S10**. Collinearity analysis between 9 NPI indicators in the Hong Kong SAR from 2020 to 2021 using the Pearson correlation method.** Table S11**. Collinearity analysis between combined NPI indicators in Beijing from 2020 to 2021 using the Pearson correlation method.** Table S12**. Collinearity analysis between combined NPI indicators in the Hong Kong SAR from 2020 to 2021 using the Pearson correlation method.** Table S13**. The goodness of fit of the GAM models with blocked cross-validation method in Beijing and the Hong Kong SAR, 2020-2021.** Table S14**. The potential impact of each individual and combined NPI on weekly ILI counts in Beijing and the Hong Kong SAR, 2020-2021*.** Table S15**. Multivariable analysis for the potential impact of NPIs on weekly ILI counts in Beijing and the Hong Kong SAR, 2020-2021*.** Table S16**. The potential impact of each individual and combined NPI on weekly ILI counts in Beijing and the Hong Kong SAR, 2020-2021*.** Table S17**. Multivariable analysis for the potential impact of NPIs on weekly ILI counts in Beijing and the Hong Kong SAR, 2020-2021*.

**Additional file 2: Figure S1**. Estimated and fitted relative reduction of weekly ILI counts by multivariate GAM model with individual NPI indicators in Beijing (a) and the Hong Kong SAR (b) in 2020–2021, based on non-COVID estimates using *Eq. **6* and data in 2011–2019. ILI: Influenza-like illness; GAM: Generalized additive model; NPI: Non-pharmaceutical intervention; Hong Kong SAR: Hong Kong Special Administrative Region; COVID: Coronavirus disease 2019.

**Additional file 3: Figure S2**. Estimated and fitted relative reduction of weekly ILI counts by multivariate GAM model with combined NPI indicators in Beijing (a) and the Hong Kong SAR (b) in 2020–2021, based on non-COVID estimates using Eq. 6 and data in 2011–2019. ILI: Influenza-like illness; GAM: Generalized additive model; NPI: Non-pharmaceutical intervention; Hong Kong SAR: Hong Kong Special Administrative Region; COVID: Coronavirus disease 2019.

**Additional file 4: Figure S3**. Estimated and fitted relative reduction of weekly ILI counts by multivariate GAM model with individual NPI indicators in Beijing (a) and the Hong Kong SAR (b) in 2020–2021, based on non-COVID estimates using Eq. 6 and data in 2011–2017. ILI: Influenza-like illness; GAM: Generalized additive model; NPI: Non-pharmaceutical intervention; Hong Kong SAR: Hong Kong Special Administrative Region; COVID: Coronavirus disease 2019.

**Additional file 5: Figure S4**. Estimated and fitted relative reduction of weekly ILI counts by multivariate GAM model with combined NPI indicators in Beijing (a) and the Hong Kong SAR (b) in 2020–2021, based on non-COVID estimates using *Eq. **6* and data in 2011–2017. ILI: Influenza-like illness; GAM: Generalized additive model; NPI: Non-pharmaceutical intervention; Hong Kong SAR: Hong Kong Special Administrative Region; COVID: Coronavirus disease 2019.

**Additional file 6: Figure S5**. Estimated and fitted relative reduction of weekly ILI counts by multivariate GAM model with individual NPI indicators in Beijing (a) and the Hong Kong SAR (b) in 2020–2021, based on non-COVID estimates using *Eq. **7* and data in 2011–2019. ILI: Influenza-like illness; GAM: Generalized additive model; NPI: Non-pharmaceutical intervention; Hong Kong SAR: Hong Kong Special Administrative Region; COVID: Coronavirus disease 2019.

**Additional file 7: Figure S6**. Estimated and fitted relative reduction of weekly ILI counts by multivariate GAM model with combined NPI indicators in Beijing (a) and the Hong Kong SAR (b) in 2020–2021, based on non-COVID estimates using *Eq. **7* and data in 2011–2019. ILI: Influenza-like illness; GAM: Generalized additive model; NPI: Non-pharmaceutical intervention; Hong Kong SAR: Hong Kong Special Administrative Region; COVID: Coronavirus disease 2019.

## Data Availability

The ILI datasets in the Hong Kong SAR analyzed during the current study are available from the Centre for Health Protection of Hong Kong SAR portal, https://www.chp.gov.hk/en/static/24015.html. The ILI datasets in Beijing analyzed during the current study are available from the corresponding authors on a reasonable request. The meteorological datasets in Beijing and the Hong Kong SAR analyzed in the current study are available from the National Centers for Environmental Information portal, https://www.ncei.noaa.gov/access/search/data-search/global-summary-of-the-day?bbox=53.544,73.620,18.198,134.761&place=Country:194&stations=54511099999&pageNum=4. The public and school holiday datasets analyzed during the current study are available from the WorldPop Data portal, https://hub.worldpop.org/project/categories?id=19. The yearly population density datasets of Beijing and the Hong Kong SAR analyzed during the current study are available from the statistical yearbook from the Beijing Municipal Bureau Statistics portal, https://nj.tjj.beijing.gov.cn/nj/main/2022-tjnj/zk/indexch.htm, and Census and Statistics Department of the Government of the Hong Kong SAR portal, https://www.censtatd.gov.hk/sc/EIndexbySubject.html?pcode=B1120017&scode=150.The R code analyzed during the current study is available from jellyzzzz-R/COVID-19-NPIs-and-seasonal-influenza (github.com).

## Abbreviations

AIC: Akaike’s information criterion
BJCDC: Beijing Center for Disease Prevention and Control
*CI*: Confidence interval
COVID-19: Coronavirus diseases 2019
Hong Kong SAR: Hong Kong Special Administrative Region
ILI: Influenza-like illness
GAM: Generalized additive model
NPIs: Non-pharmaceutical interventions
OxCGRT: Oxford COVID-19 Government Response Tracker
RMSE: Root mean squared error
SARS-CoV-2: Severe acute respiratory syndrome coronavirus 2
