# Supplementary material for: Assessing the impact of COVID-19 interventions on influenza-like illness in Beijing and Hong Kong: an observational and modeling study
Source: Infect Dis Poverty. 2023 Feb 16;12:11. doi: 10.1186/s40249-023-01061-8 (PMC9933034; doi:10.1186/s40249-023-01061-8)
Supplement: Supplementary file 1 — Additional file 1: Table S1. Individual COVID-19 NPI indicators, definition, and coding in Beijing and the Hong Kong SAR. Table S2. Combined COVID-19 NPI indicators, definition, and coding in Beijing and the Hong Kong SAR. Table S3. Collinearity analysis between meteorological factors in Beijing from 2011 to 2019 using the Pearson correlation method. Table S4. Collinearity analysis between meteorological factors in the Hong Kong SAR from 2011 to 2019 using the Pearson correlation method. Table S5. The goodness of fit of the predictive GAM models in Beijing with blocked cross-validation method. Table S6. The goodness of fit of the predictive GAM models in the Hong Kong SAR with blocked cross-validation method. Table S7. Collinearity analysis between relative change of meteorological factors in Beijing from 2020 to 2021 using the Pearson correlation method. Table S8. Collinearity analysis between relative change of meteorological factors in the Hong Kong SAR from 2020 to 2021 using the Pearson correlation method. Table S9. Collinearity analysis between 9 NPIs indicators in Beijing from 2020 to 2021 using the Pearson correlation method. Table S10. Collinearity analysis between 9 NPI indicators in the Hong Kong SAR from 2020 to 2021 using the Pearson correlation method. Table S11. Collinearity analysis between combined NPI indicators in Beijing from 2020 to 2021 using the Pearson correlation method. Table S12. Collinearity analysis between combined NPI indicators in the Hong Kong SAR from 2020 to 2021 using the Pearson correlation method. Table S13. The goodness of fit of the GAM models with blocked cross-validation method in Beijing and the Hong Kong SAR, 2020-2021. Table S14. The potential impact of each individual and combined NPI on weekly ILI counts in Beijing and the Hong Kong SAR, 2020-2021*. Table S15. Multivariable analysis for the potential impact of NPIs on weekly ILI counts in Beijing and the Hong Kong SAR, 2020-2021*. Table S16. The potential impact of each [file 40249_2023_1061_MOESM1_ESM.docx]

| **Additional Table 1**. Individual COVID-19 NPI indicators, definition, and coding in Beijing and the Hong Kong SAR | | |
| --- | --- | --- |
| **Indicators** | **Definitions** | **Coding** |
| C1 | Record closings of schools and universities | 0 - no measures  1 - recommend closing or all schools open with alterations resulting in significant differences compared to non-COVID-19 operations  2 - require closing (only some levels or categories, e.g., just high school or just public schools)  3 - require closing all levels  Blank - no data |
| C2 | Record closings of workplaces | 0 - no measures  1 - recommend closing (or recommend work from home) or all businesses open with alterations resulting in significant differences compared to non-COVID-19 operation  2 - require closing (or work from home) for some sectors or categories of workers  3 - require closing (or work from home) for all-but-essential workplaces (e.g., grocery and doctors)  Blank - no data |
| C3 | Record cancelling public events | 0 - no measures  1 - recommend cancelling  2 - require cancelling  Blank - no data |
| C4 | Record limits on gatherings | 0 - no restrictions  1 - restrictions on very large gatherings (the limit is above 1000 people)  2 - restrictions on gatherings between 101 and 1000 people  3 - restrictions on gatherings between 11-100 people  4 - restrictions on gatherings of 10 people or less  Blank - no data |
| C5 | Record closing of public transport | 0 - no measures  1 - recommend closing (or significantly reduce volume/route/means of transport available)  2 - require closing (or prohibit most citizens from using it)  Blank - no data |
| C6 | Record orders to "shelter-in-place" and otherwise confine to the home | 0 - no measures  1 - recommend not leaving house  2 - require not leaving house with exceptions for daily exercise, grocery shopping, and “essential” trips  3 - require not leaving house with minimal exceptions (e.g., allowed to leave once a week, or only one person can leave at a time, etc.)  Blank - no data |
| C7 | Record restrictions on internal movement between cities/regions | 0 - no measures  1 - recommend not to travel between regions/cities  2 - internal movement restrictions in place  Blank - no data |
| C8 | Record restrictions on international travel | 0 - no restrictions  1 - screening arrivals  2 - quarantine arrivals from some or all regions  3 - ban arrivals from some regions  4 - ban on all regions or total border closure  Blank - no data |
| H6 | Record policies on the use of facial coverings outside the home | 0 - No policy  1 - Recommended  2 - Required in some specified shared/public spaces outside the home with other people present, or some situations when social distancing not possible  3 - Required in all shared/public spaces outside the home with other people present or all situations when social distancing not possible  4 - Required outside the home at all times regardless of location or presence of other people |

| **Additional Table 2**. Combined COVID-19 NPI indicators, definition, and coding in Beijing and the Hong Kong SAR | | |
| --- | --- | --- |
| **Combined indicators** | **Definitions** | **Coding** |
| C12 | Record closings of schools or workplaces | 0 - no measures 1 - recommend closing  2 - require closing only some levels or categories 3 - require closing all levels of schools and for all-but-essential workplaces Blank - no data |
| C34 | Record cancelling public events or gatherings | 0 - no restrictions 1 - restrictions on very large public events or gatherings  2 - restrictions on medium scale public events or gatherings 3 - restrictions on small scale public events or gatherings 4 - restrictions on all public events or gatherings  Blank - no data |
| C567 | Record restrictions on internal travel | 0 - no measures 1 - recommend not to travel between regions 2 - internal movement restrictions in place 3 - require not leaving house with minimal exceptions Blank - no data |

| **Additional Table** 3. Collinearity analysis between meteorological factors in Beijing from 2011 to 2019 using the Pearson correlation method | | | |
| --- | --- | --- | --- |
|  | **Mean temperature** | **Relative humidity** | **Absolute humidity** |
| **Mean temperature** | 1.000 | 0.482 | 0.874 |
| **Relative humidity** | 0.482 | 1.000 | 0.761 |
| **Absolute humidity** | 0.874 | 0.761 | 1.000 |

| **Additional Table** 4. Collinearity analysis between meteorological factors in the Hong Kong SAR from 2011 to 2019 using the Pearson correlation method | | | |
| --- | --- | --- | --- |
|  | **Mean temperature** | **Relative humidity** | **Absolute humidity** |
| **Mean temperature** | 1.000 | 0.306 | 0.943 |
| **Relative humidity** | 0.306 | 1.000 | 0.573 |
| **Absolute humidity** | 0.943 | 0.573 | 1.000 |

| **Additional Table** 5. the goodness of fit of the predictive GAM models in Beijing with blocked cross-validation method | | | |
| --- | --- | --- | --- |
|  | RMSE | AIC | Adjusted R-squared |
| Model 1 | 0.358 (0.349, 0.367) | -193. 874 (-197.573, -190.176) | 0.697 (0.694, 0.700) |
| Model 2 | 0.243 (0.211, 0.274) | -161.519 (-165.937, -157.101) | 0.687 (0.683, 0.691) |
| Model 3 | 0.301 (0.296, 0.307) | -155.201 (-156.565, -153.837) | 0.653 (0.647, 0.659) |

Model 1 and model 2 is the same as follows and using data in 2011-2019 and 2011-2017, respectively.

$$\begin{aligned} \log\left[ E\left( Y_{i} \right) \right]=\alpha+ ns\left( {Year}_{i}, df \right)+ns\left( W_{i}, df \right) + ns\left( T_{i}, df \right) \\ +ns\left( {RH}_{i},df \right) + factor\left( H_{i} \right)+pd\#\left( 1 \right) \end{aligned}$$

Model 3 is as follows and using data in 2011-2019:

$$\begin{aligned} \log\left[ E\left( Y_{i} \right) \right]=\alpha+ns\left( W_{i}, df \right) + ns\left( T_{i}, df \right) \\ +ns\left( {RH}_{i},df \right) + factor\left( H_{i} \right)+pd\#\left( 2 \right) \end{aligned}$$

| **Additional Table** 6. the goodness of fit of the predictive GAM models in the Hong Kong SAR with blocked cross-validation method | | | |
| --- | --- | --- | --- |
|  | RMSE | AIC | Adjusted R-squared |
| Model 1 | 0.312 (0.304, 0.320) | -170.099 (-179.431, -160.766) | 0.386 (0.376, 0.396) |
| Model 2 | 0.251 (0.242, 0.261) | -188.277 (-191.907, -184.647) | 0.460 (0.453, 0.466) |
| Model 3 | 0.358 (0.344, 0.372) | -139.764 (-150.306, -129.222) | 0.318 (0.303, 0.333) |

Model 1 and model 2 is the same as follows and using data in 2011-2019 and 2011-2017, respectively.

$$\begin{aligned} \log\left[ E\left( Y_{i} \right) \right]=\alpha+ ns\left( {Year}_{i}, df \right)+ns\left( W_{i}, df \right) + ns\left( T_{i}, df \right) \\ +ns\left( {RH}_{i},df \right) + factor\left( H_{i} \right)+pd\#\left( 1 \right) \end{aligned}$$

Model 3 is as follows and using data in 2011-2019:

$$\begin{aligned} \log\left[ E\left( Y_{i} \right) \right]=\alpha+ns\left( W_{i}, df \right) + ns\left( T_{i}, df \right) \\ +ns\left( {RH}_{i},df \right) + factor\left( H_{i} \right)+pd\#\left( 2 \right) \end{aligned}$$

| **Additional Table** 7. Collinearity analysis between relative change of meteorological factors in Beijing from 2020 to 2021 using the Pearson correlation method | | | |
| --- | --- | --- | --- |
|  | **Mean temperature change** | **Relative humidity change** | **Absolute humidity change** |
| **Mean temperature change** | 1.000 | 0.102 | 0.100 |
| **Relative humidity change** | 0.102 | 1.000 | 0.901 |
| **Absolute humidity change** | 0.100 | 0.901 | 1.000 |

| **Additional Table** 8. Collinearity analysis between relative change of meteorological factors in the Hong Kong SAR from 2020 to 2021 using the Pearson correlation method | | | |
| --- | --- | --- | --- |
|  | **Mean temperature change** | **Relative humidity change** | **Absolute humidity change** |
| **Mean temperature change** | 1.000 | 0.258 | 0.716 |
| **Relative humidity change** | 0.258 | 1.000 | 0.850 |
| **Absolute humidity change** | 0.716 | 0.850 | 1.000 |

| **Additional Table 9**. Collinearity analysis between 9 NPIs indicators in Beijing from 2020 to 2021 using the Pearson correlation method | | | | | | | | | |
| --- | --- | --- | --- | --- | --- | --- | --- | --- | --- |
|  | **C1** | **C2** | **C3** | **C4** | **C5** | **C6** | **C7** | **C8** | **H6** |
| **C1** | 1.000 | 0.604 | 0.521 | 0.070 | 0.506 | 0.683 | 0.537 | -0.044 | 0.711 |
| **C2** | 0.604 | 1.000 | 0.864 | 0.320 | 0.767 | 0.345 | 0.431 | -0.068 | 0.451 |
| **C3** | 0.521 | 0.864 | 1.000 | 0.356 | 0.796 | 0.315 | 0.483 | -0.034 | 0.482 |
| **C4** | 0.070 | 0.320 | 0.356 | 1.000 | 0.066 | -0.204 | 0.509 | -0.382 | -0.104 |
| **C5** | 0.506 | 0.767 | 0.796 | 0.066 | 1.000 | 0.367 | 0.247 | -0.008 | 0.514 |
| **C6** | 0.683 | 0.345 | 0.315 | -0.204 | 0.367 | 1.000 | 0.326 | 0.231 | 0.661 |
| **C7** | 0.537 | 0.431 | 0.483 | 0.509 | 0.247 | 0.326 | 1.000 | -0.098 | 0.319 |
| **C8** | -0.044 | -0.068 | -0.034 | -0.382 | -0.008 | 0.231 | -0.098 | 1.000 | 0.099 |
| **H6** | 0.711 | 0.451 | 0.482 | -0.104 | 0.514 | 0.661 | 0.319 | 0.099 | 1.000 |

C1, C2, C3, C4, C5, C6, C7, C8, and H6 are the NPI indicators of “closings of schools and universities”, “closings of workplaces”, “cancelling public events”, “limits on gatherings”, “closing of public transport”, “shelter-in-place and otherwise confine to the home”, “restrictions on internal movement between cities/regions”, “restrictions on international travel”, and “use of facial coverings outside the home”, respectively.

| **Additional Table 10**. Collinearity analysis between 9 NPI indicators in the Hong Kong SAR from 2020 to 2021 using the Pearson correlation method | | | | | | | | | |
| --- | --- | --- | --- | --- | --- | --- | --- | --- | --- |
|  | **C1** | **C2** | **C3** | **C4** | **C5** | **C6** | **C7** | **C8** | **H6** |
| **C1** | 1.000 | 0.309 | 0.487 | -0.287 | -0.514 | 0.247 | -0.487 | 0.540 | -0.243 |
| **C2** | 0.309 | 1.000 | 0.415 | 0.319 | 0.393 | 0.326 | 0.425 | -0.133 | 0.412 |
| **C3** | 0.487 | 0.415 | 1.000 | 0.049 | -0.149 | -0.131 | -0.182 | 0.404 | 0.137 |
| **C4** | -0.287 | 0.319 | 0.049 | 1.000 | 0.442 | 0.047 | 0.411 | 0.110 | 0.618 |
| **C5** | -0.514 | 0.393 | -0.149 | 0.442 | 1.000 | 0.306 | 0.949 | -0.691 | 0.468 |
| **C6** | 0.247 | 0.326 | -0.131 | 0.047 | 0.306 | 1.000 | 0.444 | -0.088 | -0.008 |
| **C7** | -0.487 | 0.425 | -0.182 | 0.411 | 0.949 | 0.444 | 1.000 | -0.611 | 0.440 |
| **C8** | 0.540 | -0.133 | 0.404 | 0.110 | -0.691 | -0.088 | -0.611 | 1.000 | -0.107 |
| **H6** | -0.243 | 0.412 | 0.137 | 0.618 | 0.468 | -0.008 | 0.440 | -0.107 | 1.000 |

Note: C1, C2, C3, C4, C5, C6, C7, C8, and H6 are the NPI indicators of “closings of schools and universities”, “closings of workplaces”, “cancelling public events”, “limits on gatherings”, “closing of public transport”, “shelter-in-place and otherwise confine to the home”, “restrictions on internal movement between cities/regions”, “restrictions on international travel”, and “use of facial coverings outside the home”, respectively.

| **Additional Table 11**. Collinearity analysis between combined NPI indicators in Beijing from 2020 to 2021 using the Pearson correlation method | | | | | |
| --- | --- | --- | --- | --- | --- |
|  | **C12** | **C34** | **C567** | **C8** | **H6** |
| **C12** | 1.000 | 0.409 | 0.830 | -0.060 | 0.668 |
| **C34** | 0.409 | 1.000 | 0.372 | -0.330 | 0.072 |
| **C567** | 0.830 | 0.372 | 1.000 | 0.060 | 0.679 |
| **C8** | -0.060 | -0.330 | 0.060 | 1.000 | 0.099 |
| **H6** | 0.668 | 0.072 | 0.679 | 0.099 | 1.000 |

Note: C12, C34, C567, C8, and H6 are the NPI indicators of “closings of schools or workplaces”, “cancelling public events or gatherings”, “restrictions on internal travel”, “restrictions on international travel”, and “use of facial coverings outside the home”, respectively.

| **Additional Table 12**. Collinearity analysis between combined NPI indicators in the Hong Kong SAR from 2020 to 2021 using the Pearson correlation method | | | | | |
| --- | --- | --- | --- | --- | --- |
|  | **C12** | **C34** | **C567** | **C8** | **H6** |
| **C12** | 1.000 | 0.202 | 0.029 | 0.282 | 0.078 |
| **C34** | 0.202 | 1.000 | 0.279 | 0.257 | 0.615 |
| **C567** | 0.029 | 0.279 | 1.000 | -0.585 | 0.387 |
| **C8** | 0.282 | 0.257 | -0.585 | 1.000 | -0.105 |
| **H6** | 0.078 | 0.615 | 0.387 | -0.105 | 1.000 |

Note: C12, C34, C567, C8, and H6 are the NPI indicators of “closings of schools or workplaces”, “cancelling public events or gatherings”, “restrictions on internal travel”, “restrictions on international travel”, and “use of facial coverings outside the home”, respectively.

| **Additional Table** 13. the goodness of fit of the GAM models with blocked cross-validation method in Beijing and the Hong Kong SAR, 2020-2021 | | | |
| --- | --- | --- | --- |
|  | **RMSE** | **AIC** | **Adjusted R-squared** |
| for Beijing |  |  |  |
| $E\left( Y_{i}\_c \right)= \alpha_{c}+ns\left( S_{i}, df \right) + ns\left( T_{i}\_c, df \right)+ ns\left( {RH}_{i}\_c,df \right) + pd\_c+C3+C4+C6+C7+C8+H6$ |  |  |  |
| Predicted by model 1 | 0.255 (0.202, 0.307) | -96.906 ( -98.833, -94.978) | 0.914 (0.909, 0.919) |
| Predicted by model 2 | 0.150 (0.119, 0.181) | -161.632 (-164.049, -159.216) | 0.936 (0.933, 0.939) |
| Predicted by model 3 | 0.178 (0.140, 0.216) | -143.099 (-145.288, -140.910) | 0.928 (0.925, 0.932) |
| $E\left( Y_{i}\_c \right)= \alpha_{c}+ns\left( S_{i}, df \right) + ns\left( T_{i}\_c, df \right)+ ns\left( {RH}_{i}\_c,df \right) + pd\_c+C34+C567+C8+H6$ |  |  |  |
| Predicted by model 1 | 0.295 (0.225, 0.365) | -94.610 (-96.595, -92.624) | 0.908 (0.903, 0.913) |
| Predicted by model 2 | 0.172 (0.134, 0.210) | -159.292 (-161.717, -156.867) | 0.931 (0.928, 0.935) |
| Predicted by model 3 | 0.197 (0.157, 0.238) | -142.169 (-144.286, -140.052) | 0.926 (0.922, 0.929) |
| for the Hong Kong SAR |  |  |  |
| $E\left( Y_{i}\_c \right)= \alpha_{c}+ns\left( S_{i}, df \right) + ns\left( T_{i}\_c, df \right)+ ns\left( {RH}_{i}\_c,df \right) + pd\_c+C1+C2+C3+C4+C6+C7+C8+H6$ |  |  |  |
| Predicted by model 1 | 0.415 (0.327, 0.504) | -92.451 (-97.248, -87.655) | 0.871 (0.857, 0.885) |
| Predicted by model 2 | 0.152 (0.113, 0.190) | -181.520 (-187.401, -175.640) | 0.900 (0.881, 0.919) |
| Predicted by model 3 | 0.217 (0.165, 0.268) | -151.258 (-157.850, -144.666) | 0.860 (0.832, 0.888) |
| $E\left( Y_{i}\_c \right)= \alpha_{c}+ns\left( S_{i}, df \right) + ns\left( T_{i}\_c, df \right)+ ns\left( {RH}_{i}\_c,df \right) + pd\_c+C12+C34+C567+C8+H6$ |  |  |  |
| Predicted by model 1 | 0.435 (0.346, 0.523) | -93.946 (-98.554, -89.336) | 0.873 (0.859, 0.886) |
| Predicted by model 2 | 0.152 (0.117, 0.186) | -180.431 (-185.986, -174.877) | 0.898 (0.879, 0.916) |
| Predicted by model 3 | 0.214 (0.169, 0.260) | -145.987 (-150.656, -141.317) | 0.850 (0.825, 0.876) |

$E\left( Y_{i}\_c \right)$is the expected relative reduction of ILI counts in week (*i)*; $\alpha_{c}$ is the intercept; $S_{i}$is a time series of week numbers (1,2,3…, 98) during the study period, representing the potential seasonality and long-term trend in weekly ILI counts; $T_{i}\_c$ is the relative change of mean temperature in week *(i)*; ${RH}_{i}\_c$ is the relative change of relative humidity in week (*i)*; $pd\_c$ is the relative change of population density. Model 1 and model 2 is the same as follows *(Eq.1)* and using data in 2011-2019 and 2011-2017, respectively.

$$\begin{aligned} \log\left[ E\left( Y_{i} \right) \right]=\alpha+ ns\left( {Year}_{i}, df \right)+ns\left( W_{i}, df \right) + ns\left( T_{i}, df \right) \\ +ns\left( {RH}_{i},df \right) + factor\left( H_{i} \right)+pd\#\left( 1 \right) \end{aligned}$$

Model 3 is as follows *(Eq.2)* and using data in 2011-2019:

$$\begin{aligned} \log\left[ E\left( Y_{i} \right) \right]=\alpha+ns\left( W_{i}, df \right) + ns\left( T_{i}, df \right) \\ +ns\left( {RH}_{i},df \right) + factor\left( H_{i} \right)+pd\#\left( 2 \right) \end{aligned}$$

| **Additional Table** 14. The potential impact of each individual and combined NPI on weekly ILI counts in Beijing and the Hong Kong SAR, 2020-2021* | | |
| --- | --- | --- |
| **Interventions** | **Beijing** | **the Hong Kong SAR** |
| C1: Closings of schools | -0.088 (-0.153, -0.022) * | 0.04 (0.005, 0.074) * |
| C2: Closings of workplaces | -0.127 (-0.178, -0.076) * | 0.037 (0.008, 0.067) * |
| C3: Cancelling public events | -0.169 (-0.218, -0.119) * | -0.006 (-0.044, 0.032) |
| C4: Limits on gatherings | -0.05 (-0.074, -0.026) * | -0.004 (-0.032, 0.025) |
| C5: Closing of public transport | -0.105 (-0.177, -0.033) * | 0.364 (0.251, 0.477) * |
| C6: Staying in place or at home | -0.082 (-0.134, -0.03) * | 0.114 (0.042, 0.185) * |
| C7: Restrictions on internal travel | -0.165 (-0.215, -0.115) * | 0.094 (-0.018, 0.207) |
| C8: Restrictions on international travel | 0.06 (0.01, 0.11) * | -0.106 (-0.146, -0.065) * |
| H6: Mask wearing outside the home | -0.162 (-0.271, -0.053) * | 0.055 (0.003, 0.107) * |
| C12: Closings of schools and workplaces | -0.167 (-0.234, -0.1) * | 0.042 (0.008, 0.076) * |
| C34: Cancelling public events or gatherings | -0.1 (-0.136, -0.065) * | -0.005 (-0.049, 0.038) |
| C567: Restrictions on internal movement | -0.086 (-0.112, -0.06) * | 0.256 (0.131, 0.38) * |
| All NPIs as a whole | -0.282 (-0.363, -0.201) * | 0.051 (-0.026, 0.127) |

*, predicted by Eq.1 using data in 2011-2019

$$\begin{aligned} \log\left[ E\left( Y_{i} \right) \right]=\alpha+ ns\left( {Year}_{i}, df \right)+ns\left( W_{i}, df \right) + ns\left( T_{i}, df \right) \\ +ns\left( {RH}_{i},df \right) + factor\left( H_{i} \right)+pd\#\left( 1 \right) \end{aligned}$$

| **Additional Table** 15. Multivariable analysis for the potential impact of NPIs on weekly ILI counts in Beijing and the Hong Kong SAR, 2020-2021* | | |
| --- | --- | --- |
| **Interventions** | **Beijing** | **the Hong Kong SAR** |
| For individual NPI indicators |  |  |
| +C1: Closings of schools | - | -0.021 (-0.069, 0.028) |
| +C2: Closings of workplaces | - | 0.056 (0.007, 0.105) * |
| +C3: Cancelling public events | -0.14 (-0.191, -0.089) * | -0.024 (-0.068, 0.019) |
| +C4: Limits on gatherings | 0.029 (-0.001, 0.059) | -0.014 (-0.044, 0.016) |
| +C5: Closing of public transport | - | - |
| +C6: Staying in place or at home | -0.032 (-0.076, 0.012) | 0.108 (-0.001, 0.216) |
| +C7: Restrictions on internal travel | -0.123 (-0.187, -0.059) * | -0.04 (-0.203, 0.124) |
| +C8: Restrictions on international travel | 0.028 (-0.013, 0.07) | -0.116 (-0.157, -0.075) * |
| +H6: Mask wearing outside the home | -0.115 (-0.204, -0.027) * | 0.008 (-0.054, 0.07) |
| For combined NPI indicators |  |  |
| +C12: Closings of schools and workplaces | - | 0.027 (-0.018, 0.072) |
| +C34: Cancelling public events or gatherings | -0.049 (-0.095, -0.004) * | -0.009 (-0.054, 0.035) |
| +C567: Restrictions on internal movement | -0.062 (-0.094, -0.03) * | 0.151 (0.012, 0.29) * |
| +C8: Restrictions on international travel | -0.006 (-0.051, 0.038) | -0.112 (-0.152, -0.071) * |
| +H6: Mask wearing outside the home | -0.082 (-0.179, 0.015) | 0.012 (-0.043, 0.066) |

*, predicted by Eq.1 using data in 2011-2019

$$\begin{aligned} \log\left[ E\left( Y_{i} \right) \right]=\alpha+ ns\left( {Year}_{i}, df \right)+ns\left( W_{i}, df \right) + ns\left( T_{i}, df \right) \\ +ns\left( {RH}_{i},df \right) + factor\left( H_{i} \right)+pd\#\left( 1 \right) \end{aligned}$$

| **Additional Table** 16. The potential impact of each individual and combined NPI on weekly ILI counts in Beijing and the Hong Kong SAR, 2020-2021* | | |
| --- | --- | --- |
| **Interventions** | **Beijing** | **the Hong Kong SAR** |
| C1: Closings of schools | -0.09 (-0.197, 0.018) | 0.109 (0.017, 0.2) * |
| C2: Closings of workplaces | -0.162 (-0.251, -0.073) * | 0.107 (0.033, 0.182) * |
| C3: Cancelling public events | -0.244 (-0.33, -0.159) * | 0.001 (-0.093, 0.096) |
| C4: Limits on gatherings | -0.058 (-0.1, -0.016) * | 0.009 (-0.062, 0.079) |
| C5: Closing of public transport | -0.133 (-0.253, -0.013) * | 0.822 (0.513, 1.132) * |
| C6: Staying in place or at home | -0.113 (-0.199, -0.026) * | 0.327 (0.133, 0.521) * |
| C7: Restrictions on internal travel | -0.227 (-0.315, -0.139) * | 0.208 (-0.075, 0.492) |
| C8: Restrictions on international travel | 0.052 (-0.031, 0.135) | -0.206 (-0.305, -0.106) * |
| H6: Mask wearing outside the home | -0.178 (-0.362, 0.006) | 0.141 (0.005, 0.276) * |
| C12: Closings of schools and workplaces | -0.2 (-0.318, -0.083) * | 0.116 (0.027, 0.206) * |
| C34: Cancelling public events or gatherings | -0.13 (-0.193, -0.067) * | 0.014 (-0.095, 0.123) |
| C567: Restrictions on internal movement | -0.123 (-0.168, -0.077) * | 0.711 (0.36, 1.063) * |
| All NPIs as a whole | -0.37 (-0.514, -0.225) * | 0.142 (-0.051, 0.336) |

*, predicted by Eq.1 using data in 2011-2017

$$\begin{aligned} \log\left[ E\left( Y_{i} \right) \right]=\alpha+ ns\left( {Year}_{i}, df \right)+ns\left( W_{i}, df \right) + ns\left( T_{i}, df \right) \\ +ns\left( {RH}_{i},df \right) + factor\left( H_{i} \right)+pd\#\left( 1 \right) \end{aligned}$$

| **Additional Table** 17. Multivariable analysis for the potential impact of NPIs on weekly ILI counts in Beijing and the Hong Kong SAR, 2020-2021* | | |
| --- | --- | --- |
| **Interventions** | **Beijing** | **the Hong Kong SAR** |
| For individual NPI indicators |  |  |
| +C1: Closings of schools | - | -0.067 (-0.202, 0.068) |
| +C2: Closings of workplaces | - | 0.155 (0.02, 0.289) * |
| +C3: Cancelling public events | -0.237 (-0.33, -0.143) * | -0.059 (-0.178, 0.061) |
| +C4: Limits on gatherings | 0.065 (0.01, 0.12) * | -0.032 (-0.114, 0.051) |
| +C5: Closing of public transport | - | - |
| +C6: Staying in place or at home | -0.078 (-0.159, 0.004) | 0.245 (-0.054, 0.545) |
| +C7: Restrictions on internal travel | -0.188 (-0.306, -0.071) * | -0.045 (-0.497, 0.407) |
| +C8: Restrictions on international travel | 0.033 (-0.044, 0.11) | -0.235 (-0.347, -0.123) * |
| +H6: Mask wearing outside the home | -0.111 (-0.272, 0.05) | 0.029 (-0.144, 0.201) |
| For combined NPI indicators |  |  |
| +C12: Closings of schools and workplaces | - | 0.093 (-0.16, 0.346) |
| +C34: Cancelling public events or gatherings | -0.05 (-0.131, 0.031) | -1.103 (-1.805, -0.4) * |
| +C567: Restrictions on internal movement | -0.099 (-0.156, -0.041) * | -0.466 (-1.027, 0.096) |
| +C8: Restrictions on international travel | -0.017 (-0.097, 0.063) | -0.232 (-0.352, -0.112) * |
| +H6: Mask wearing outside the home | -0.078 (-0.249, 0.093) | 1.621 (0.707, 2.535) * |

*, predicted by Eq.1 using data in 2011-2017

$$\begin{aligned} \log\left[ E\left( Y_{i} \right) \right]=\alpha+ ns\left( {Year}_{i}, df \right)+ns\left( W_{i}, df \right) + ns\left( T_{i}, df \right) \\ +ns\left( {RH}_{i},df \right) + factor\left( H_{i} \right)+pd\#\left( 1 \right) \end{aligned}$$
