# Supplementary figures and images for: Assessing the impact of COVID-19 interventions on influenza-like illness in Beijing and Hong Kong: an observational and modeling study
Source: Infect Dis Poverty. 2023 Feb 16;12:11. doi: 10.1186/s40249-023-01061-8 (PMC9933034; doi:10.1186/s40249-023-01061-8)

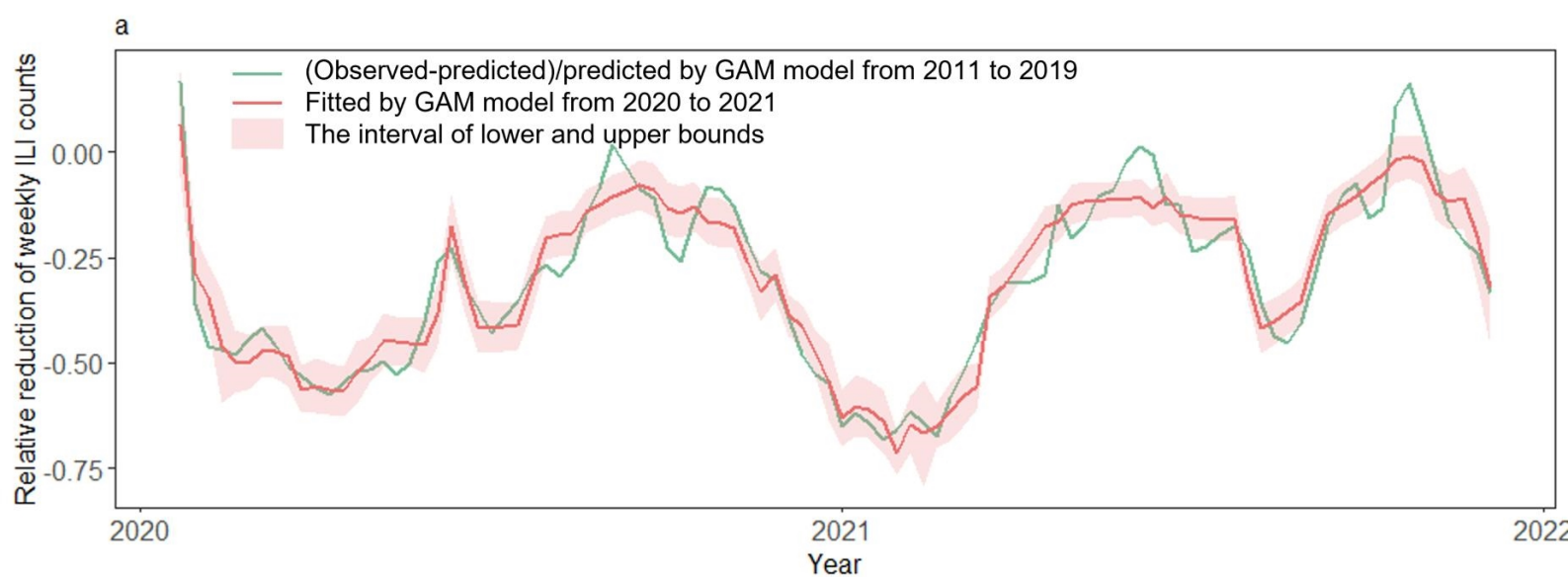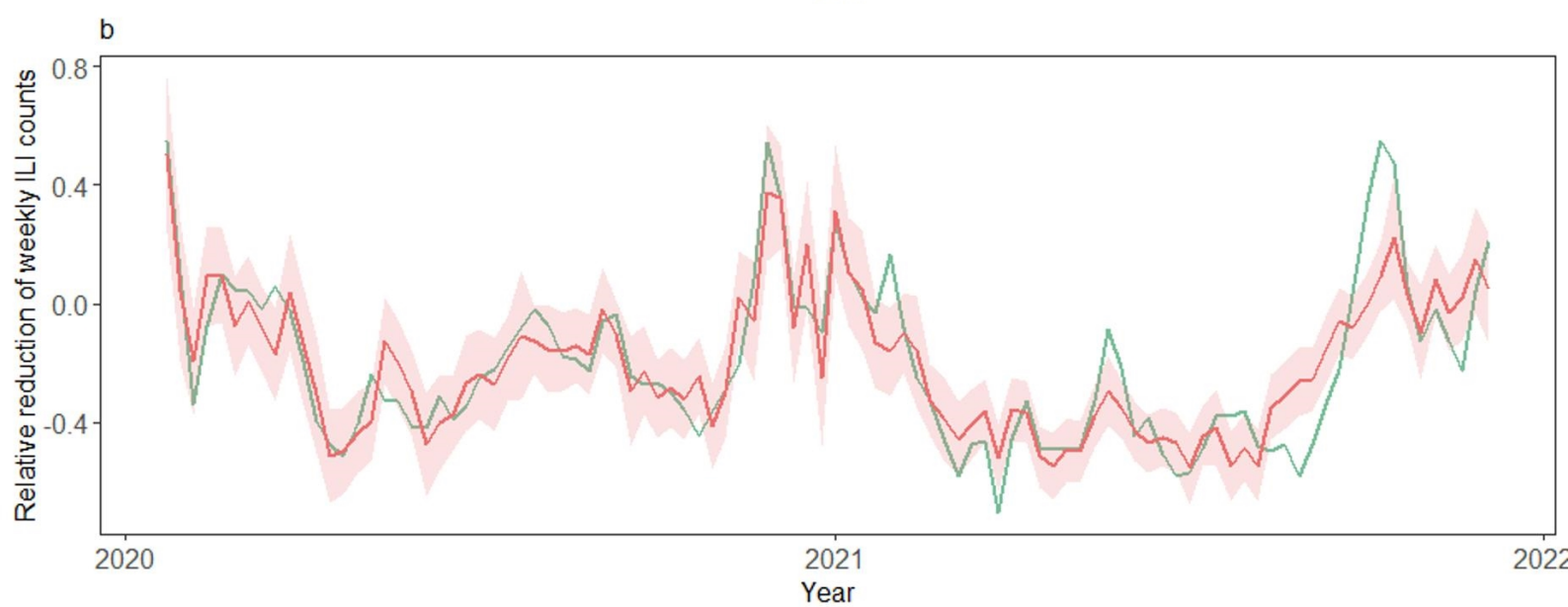

Supplement: Supplementary file 2 — Additional file 2: Figure S1. Estimated and fitted relative reduction of weekly ILI counts by multivariate GAM model with individual NPI indicators in Beijing (a) and the Hong Kong SAR (b) in 2020–2021, based on non-COVID estimates using Eq. 6 and data in 2011–2019. ILI: Influenza-like illness; GAM: Generalized additive model; NPI: Non-pharmaceutical intervention; Hong Kong SAR: Hong Kong Special Administrative Region; COVID: Coronavirus disease 2019. [file 40249_2023_1061_MOESM2_ESM.pdf]

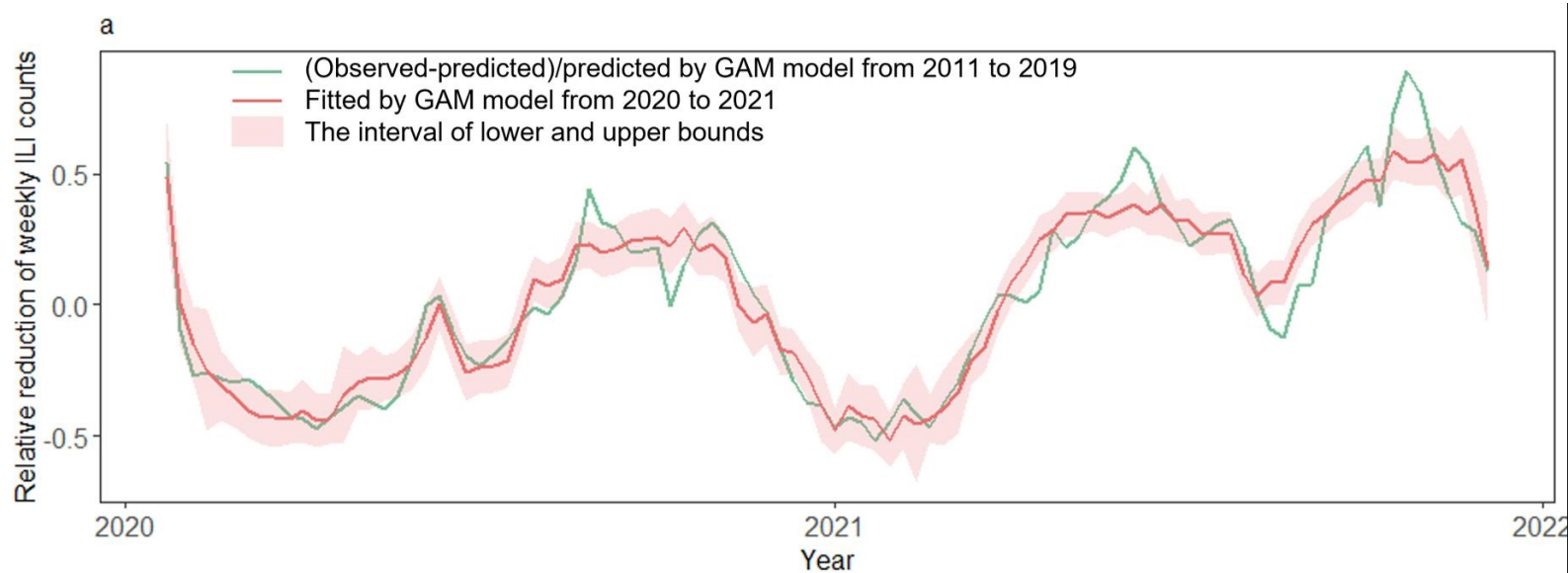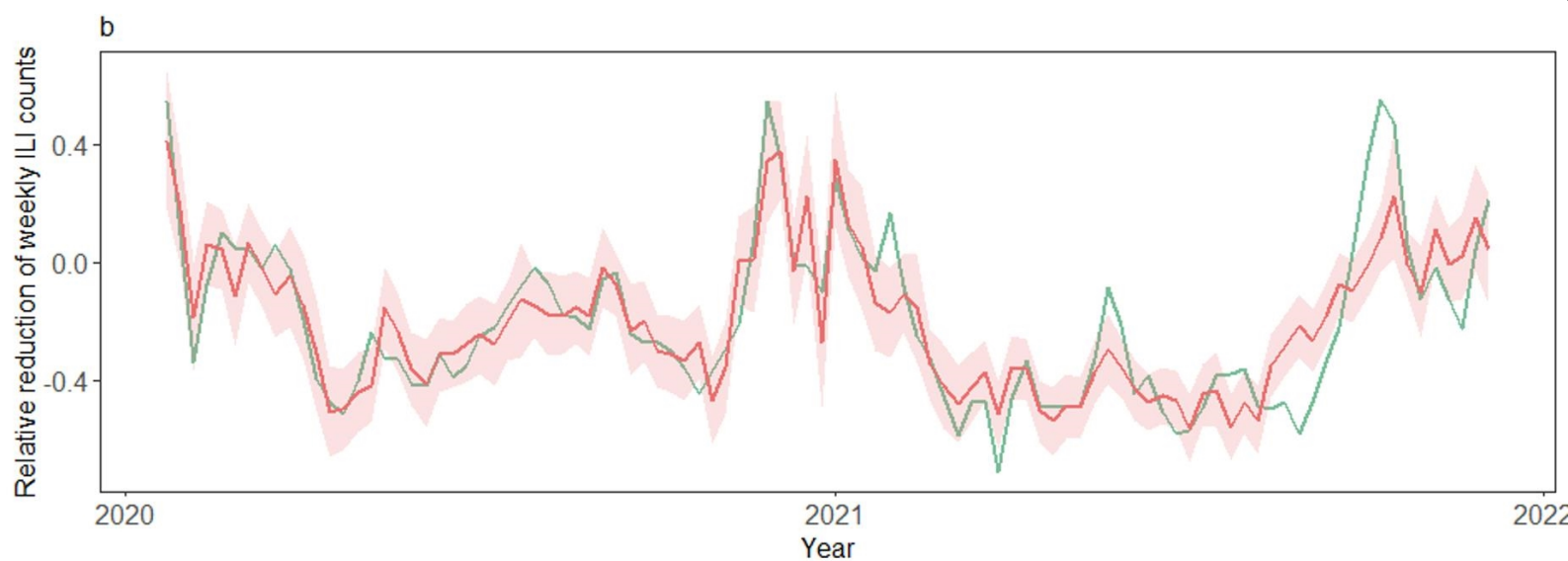

Supplement: Supplementary file 3 — Additional file 3: Figure S2. Estimated and fitted relative reduction of weekly ILI counts by multivariate GAM model with combined NPI indicators in Beijing (a) and the Hong Kong SAR (b) in 2020–2021, based on non-COVID estimates using Eq. 6 and data in 2011–2019. ILI: Influenza-like illness; GAM: Generalized additive model; NPI: Non-pharmaceutical intervention; Hong Kong SAR: Hong Kong Special Administrative Region; COVID: Coronavirus disease 2019. [file 40249_2023_1061_MOESM3_ESM.pdf]

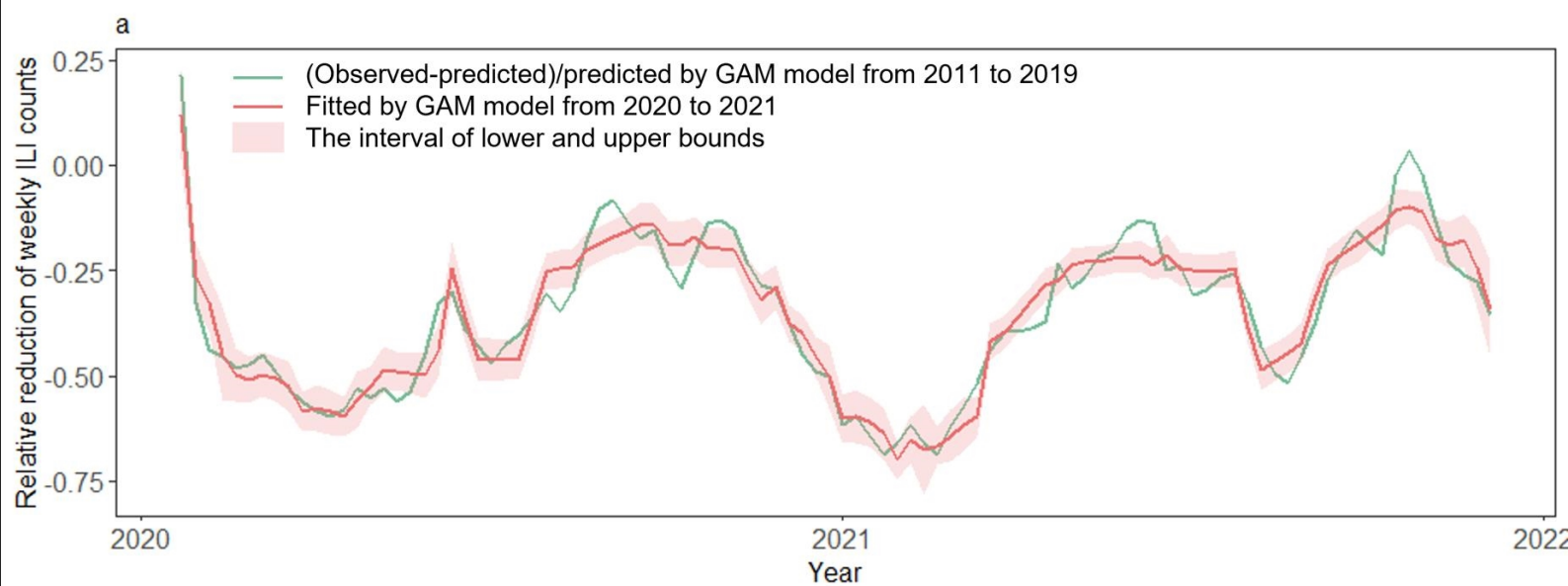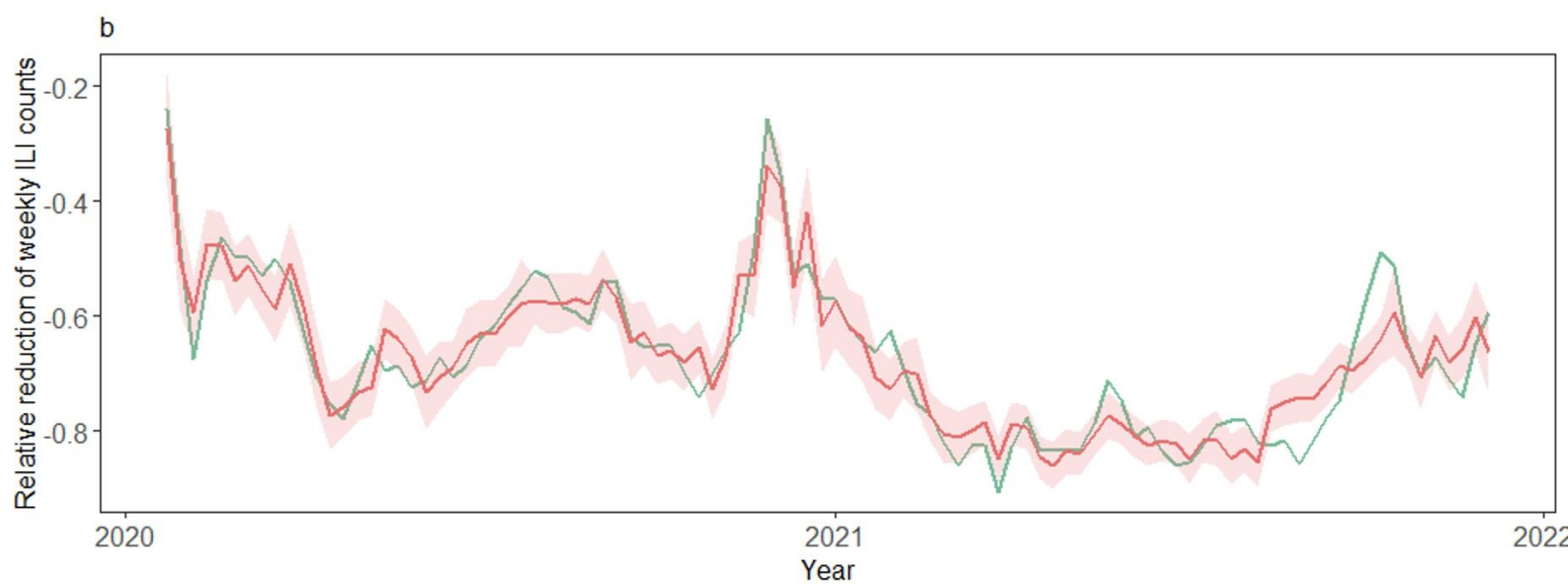

Supplement: Supplementary file 4 — Additional file 4: Figure S3. Estimated and fitted relative reduction of weekly ILI counts by multivariate GAM model with individual NPI indicators in Beijing (a) and the Hong Kong SAR (b) in 2020–2021, based on non-COVID estimates using Eq. 6 and data in 2011–2017. ILI: Influenza-like illness; GAM: Generalized additive model; NPI: Non-pharmaceutical intervention; Hong Kong SAR: Hong Kong Special Administrative Region; COVID: Coronavirus disease 2019. [file 40249_2023_1061_MOESM4_ESM.pdf]

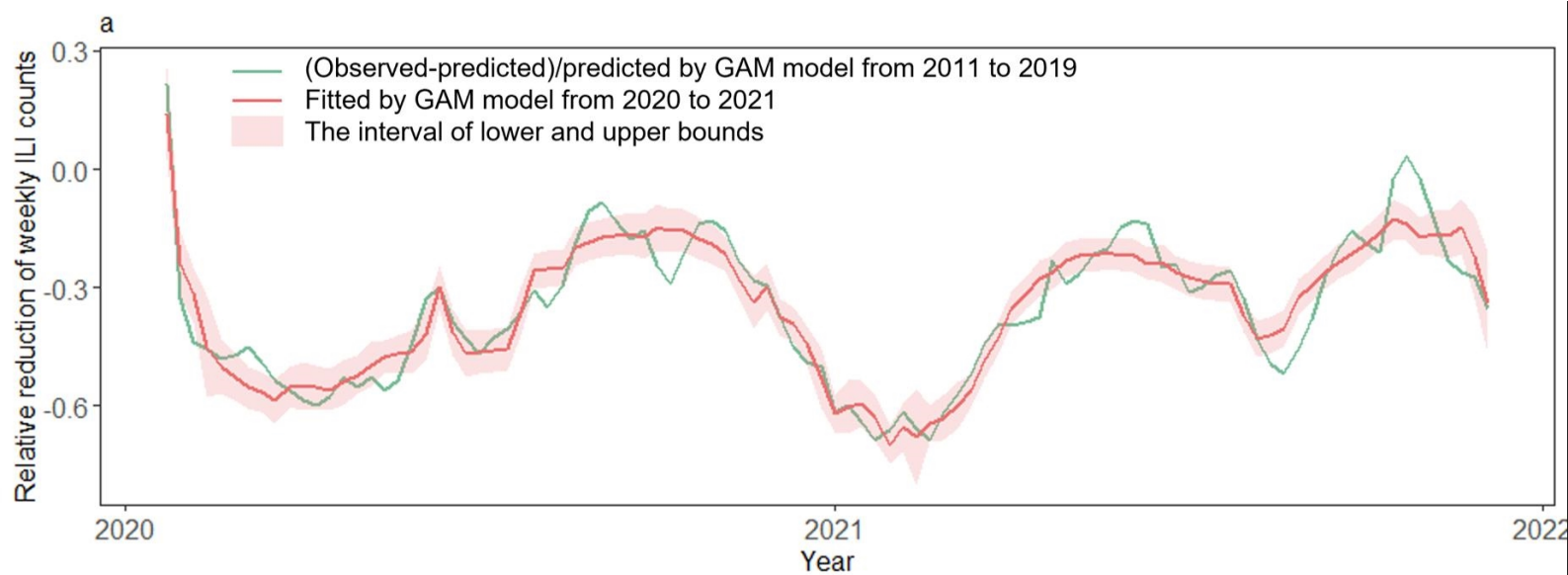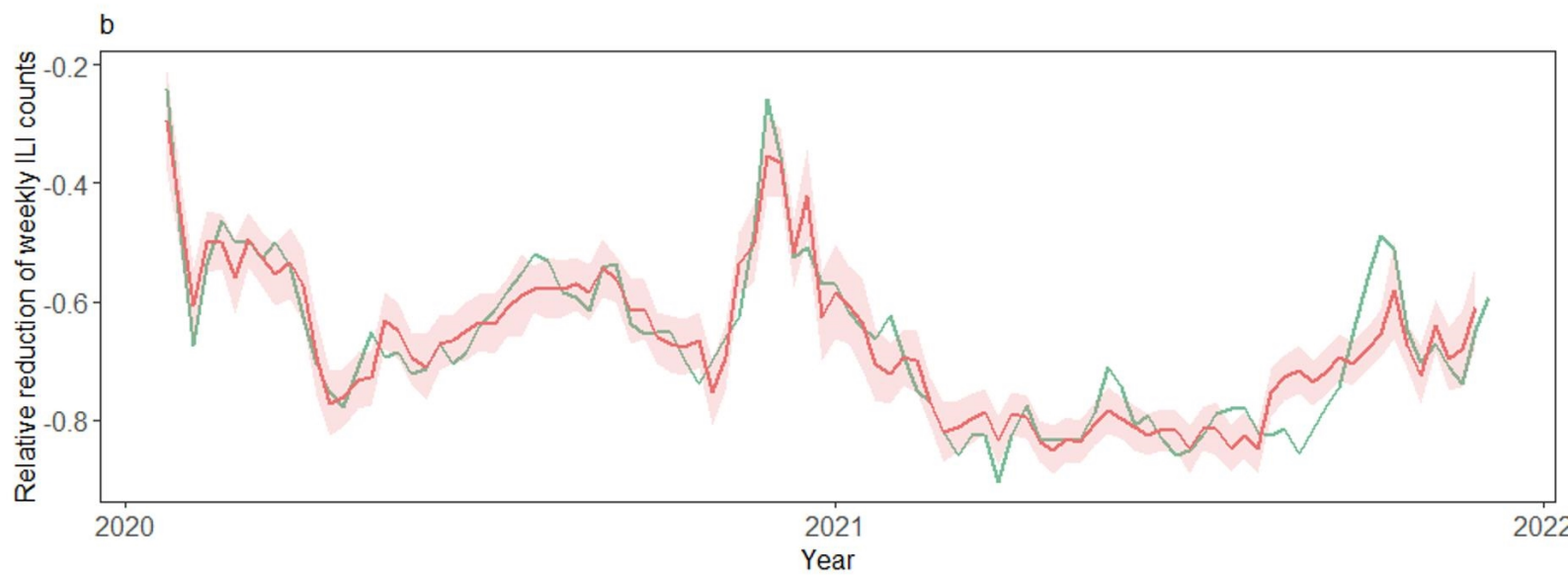

Supplement: Supplementary file 5 — Additional file 5: Figure S4. Estimated and fitted relative reduction of weekly ILI counts by multivariate GAM model with combined NPI indicators in Beijing (a) and the Hong Kong SAR (b) in 2020–2021, based on non-COVID estimates using Eq. 6 and data in 2011–2017. ILI: Influenza-like illness; GAM: Generalized additive model; NPI: Non-pharmaceutical intervention; Hong Kong SAR: Hong Kong Special Administrative Region; COVID: Coronavirus disease 2019. [file 40249_2023_1061_MOESM5_ESM.pdf]

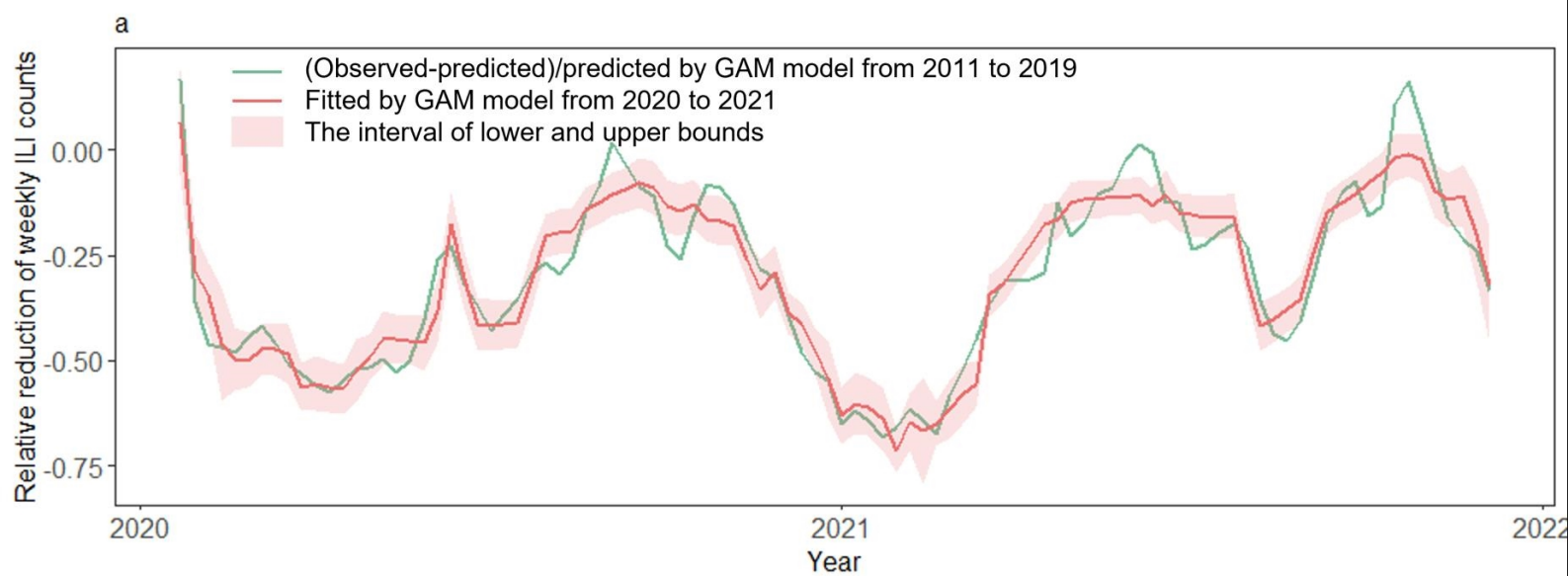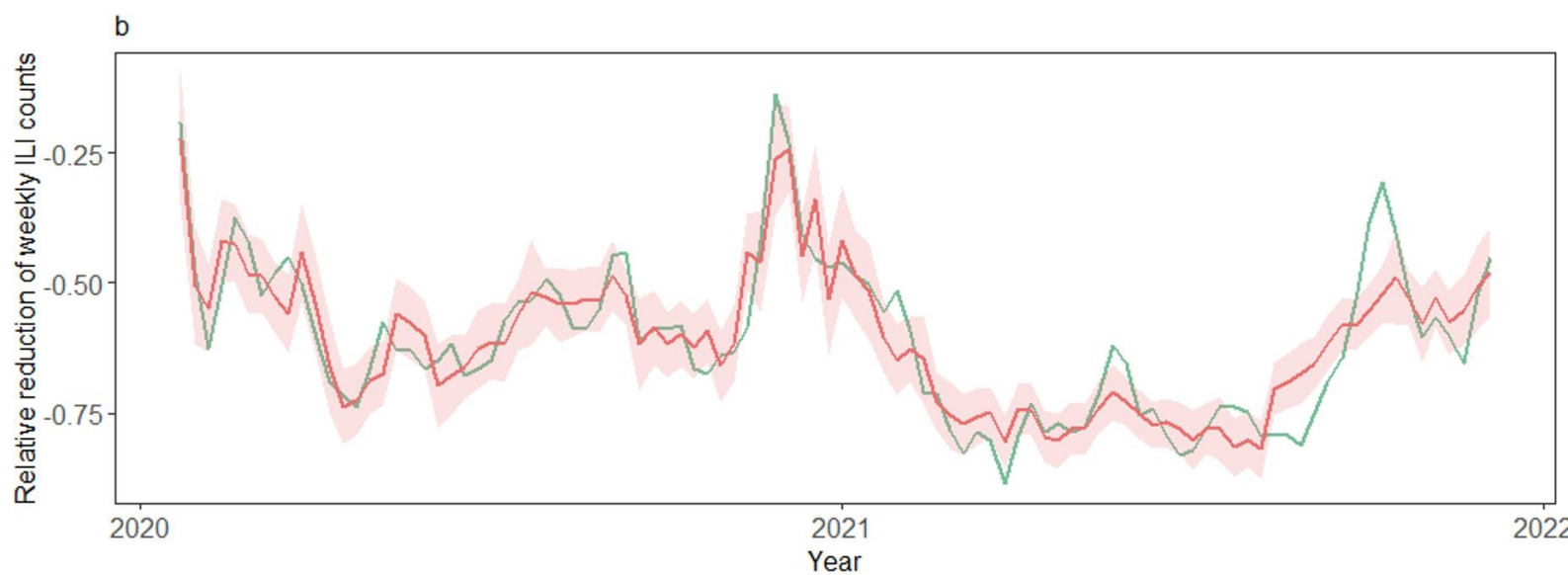

Supplement: Supplementary file 6 — Additional file 6: Figure S5. Estimated and fitted relative reduction of weekly ILI counts by multivariate GAM model with individual NPI indicators in Beijing (a) and the Hong Kong SAR (b) in 2020–2021, based on non-COVID estimates using Eq. 7 and data in 2011–2019. ILI: Influenza-like illness; GAM: Generalized additive model; NPI: Non-pharmaceutical intervention; Hong Kong SAR: Hong Kong Special Administrative Region; COVID: Coronavirus disease 2019. [file 40249_2023_1061_MOESM6_ESM.pdf]

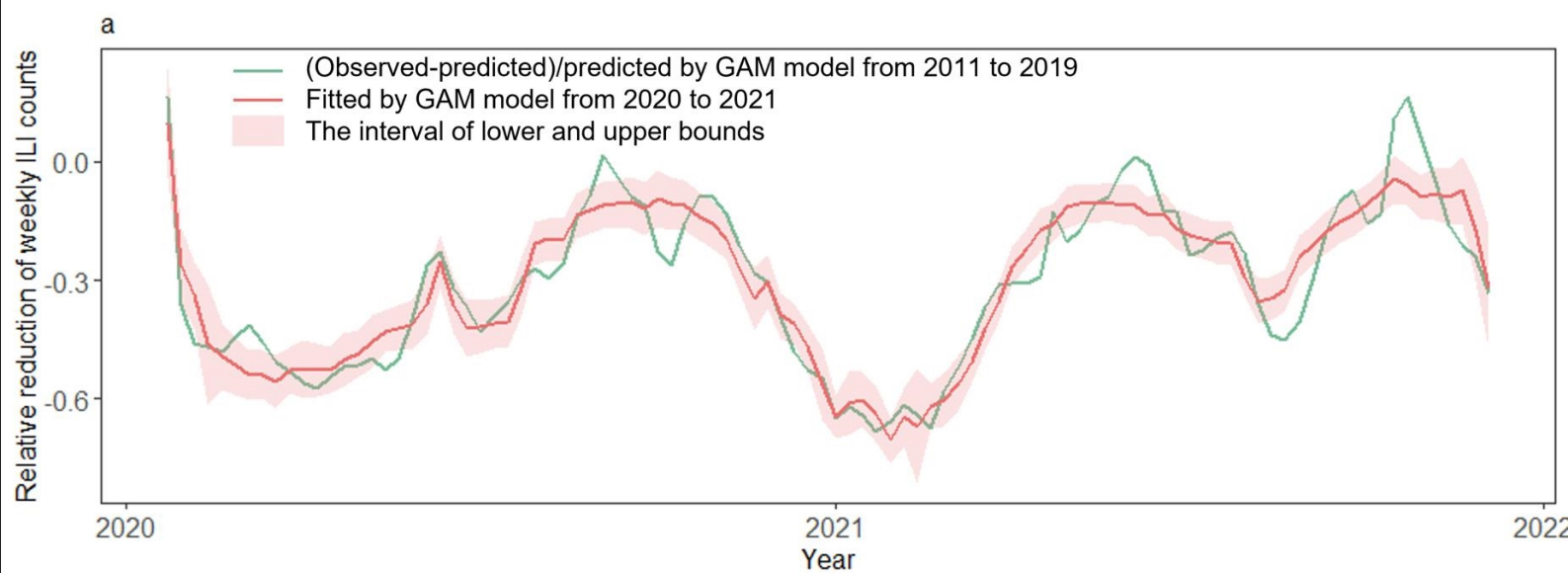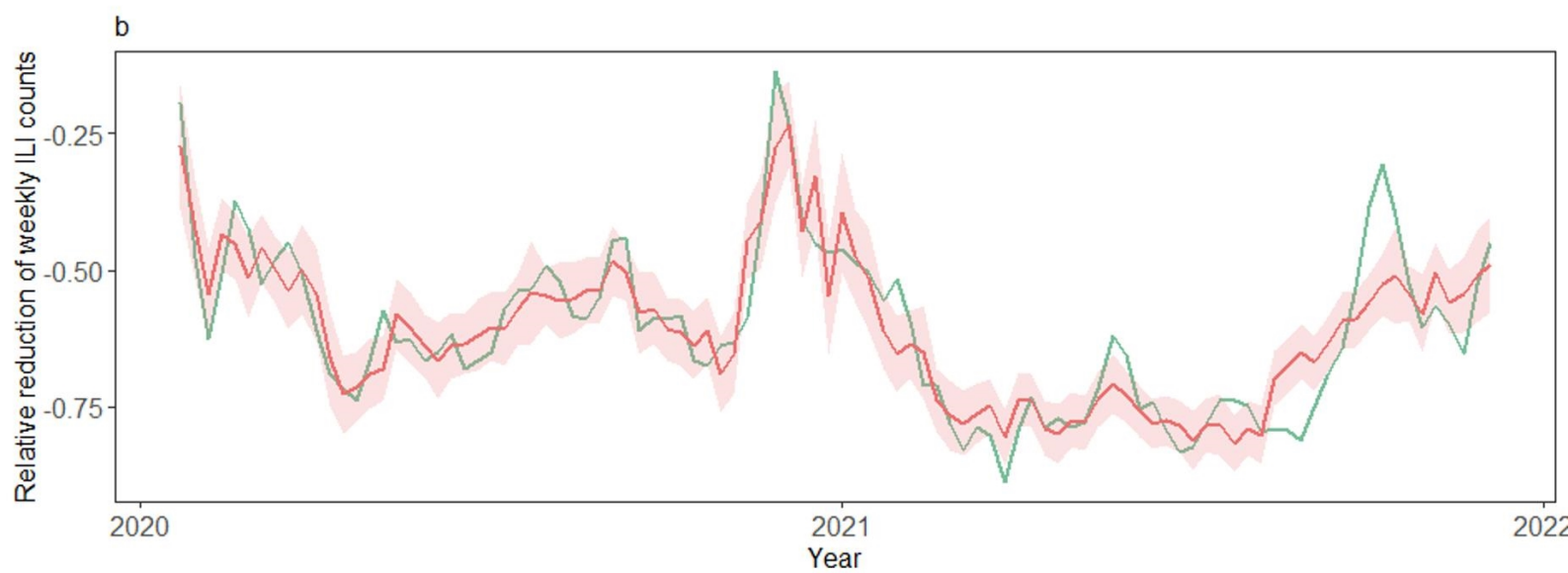

Supplement: Supplementary file 7 — Additional file 7: Figure S6. Estimated and fitted relative reduction of weekly ILI counts by multivariate GAM model with combined NPI indicators in Beijing (a) and the Hong Kong SAR (b) in 2020–2021, based on non-COVID estimates using Eq. 7 and data in 2011–2019. ILI: Influenza-like illness; GAM: Generalized additive model; NPI: Non-pharmaceutical intervention; Hong Kong SAR: Hong Kong Special Administrative Region; COVID: Coronavirus disease 2019. [file 40249_2023_1061_MOESM7_ESM.pdf]
